# Relativistic spacetime crystals

**DOI:** 10.1107/S2053273321003259

**Published:** 2021-05-27

**Authors:** Venkatraman Gopalan

**Affiliations:** a Department of Materials Science and Engineering, Department of Physics, Department of Engineering Science and Mechanics, and the Materials Research Institute, Pennsylvania State University, University Park, PA 16802, USA

**Keywords:** spacetime, special relativity, renormalized blended spacetime, relativistic spacetime crystals

## Abstract

Periodic space crystals are well established and widely used in physical sciences. Time crystals have been increasingly explored more recently, where time is disconnected from space. Periodic relativistic spacetime crystals on the other hand need to account for the mixing of space and time in special relativity through Lorentz transformation, and have been listed only in 2D. This work shows that there exists a transformation between the conventional Minkowski spacetime (MS) and what is referred to here as renormalized blended spacetime (RBS); they are shown to be equivalent descriptions of relativistic physics in flat spacetime. There are two elements to this reformulation of MS, namely, blending and renormalization. When observers in two inertial frames adopt each other’s clocks as their own, while retaining their original space coordinates, the observers become blended. This process reformulates the Lorentz boosts into Euclidean rotations while retaining the original spacetime hyperbola describing worldlines of constant spacetime length from the origin. By renormalizing the blended coordinates with an appropriate factor that is a function of the relative velocities between the various frames, the hyperbola is transformed into a Euclidean circle. With these two steps, one obtains the RBS coordinates complete with new light lines, but now with a Euclidean construction. One can now enumerate the RBS point and space groups in various dimensions with their mapping to the well known space crystal groups. The RBS point group for flat isotropic RBS spacetime is identified to be that of cylinders in various dimensions: ***mm*2** which is that of a rectangle in 2D, (∞/***m***) ***m*** which is that of a cylinder in 3D, and that of a hypercylinder in 4D. An antisymmetry operation is introduced that can swap between space-like and time-like directions, leading to color spacetime groups. The formalism reveals RBS symmetries that are not readily apparent in the conventional MS formulation. *Mathematica* script is provided for plotting the MS and RBS geometries discussed in the work.

## Abstract 2

By appropriate reformulation of relativistic spacetime geometry, a direct mapping to Euclidean space crystals is shown. Using this mapping, hidden symmetries in relativistic spacetime crystals are uncovered.

## 1. Minkowski spacetime (MS), (*x, ct*)   

The goal of this work is to illustrate a transformation between the conventional flat relativistic spacetime (also called the Minkowski spacetime, MS, whose geometry is hyperbolic) and what is referred to here as renormalized blended spacetime (RBS, whose geometry is Euclidean). This will then form the basis for a mapping of the RBS crystals to the well known space crystals, which in turn will help enumerate the former. To achieve this, we first briefly introduce the MS, followed by two critical steps required to reformulate it into RBS, namely, blending and renormalization. The former will largely retain the structure of the MS except to describe it with Euclidean angles and functions instead of hyperbolic angles and functions. The latter will transform the hyperbola into a circle. We largely adopt a geometric approach to special relativity and work in the early sections with 2D spacetime to keep the treatment accessible.

The geometry of a Euclidean 2D space spanned by unit vectors **x** and **y** possesses a norm (square) that is positive, *i.e.* 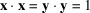 . (Bold font is used for vectors and roman font for coordinates.) In 2D space, the length *r* of a vector **r** from the origin to a point *P* is invariant under linear orthogonal transformations such as Euclidean rotations, inversion or mirror. Given the coordinates (*x, y*) of the point *P* in the unprimed Euclidean coordinate system, and ( 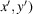 in the primed Euclidean coordinate system that shares the same origin and is related to the unprimed coordinate system by a linear orthogonal transformation, the length of the vector *r* will remain invariant, *i.e.*


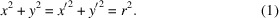




**Figure 1:**
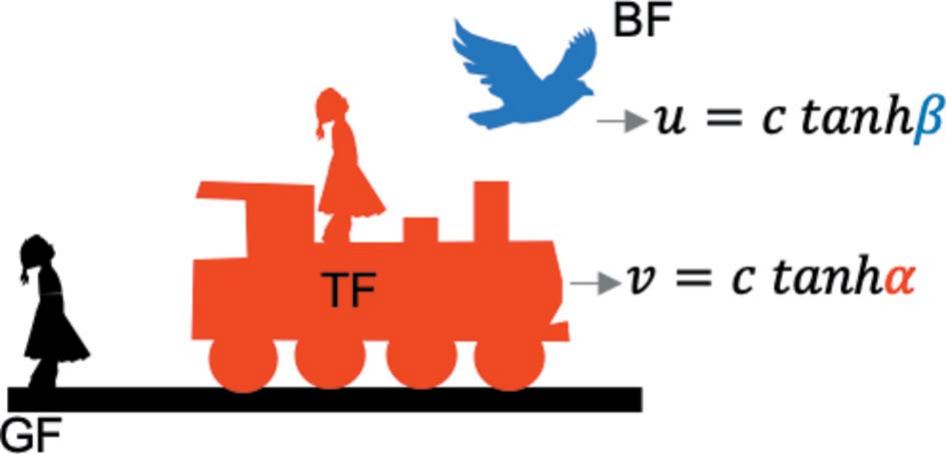
The schematic depicts the stationary ground frame (GF, 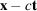 ) observer. With respect to the GF, the train frame (TF, 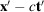 ) observer moves with a velocity *v* in the 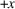 direction. With respect to the GF, an event (a bird) frame (BF) moves at a velocity *u* in the 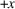 direction. The hyperbolic angles ( 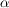 and 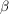 ) are defined by the velocities *u* and *v* relative to *c* as indicated, and are illustrated in Fig. 2 ▸.

**Figure 2:**
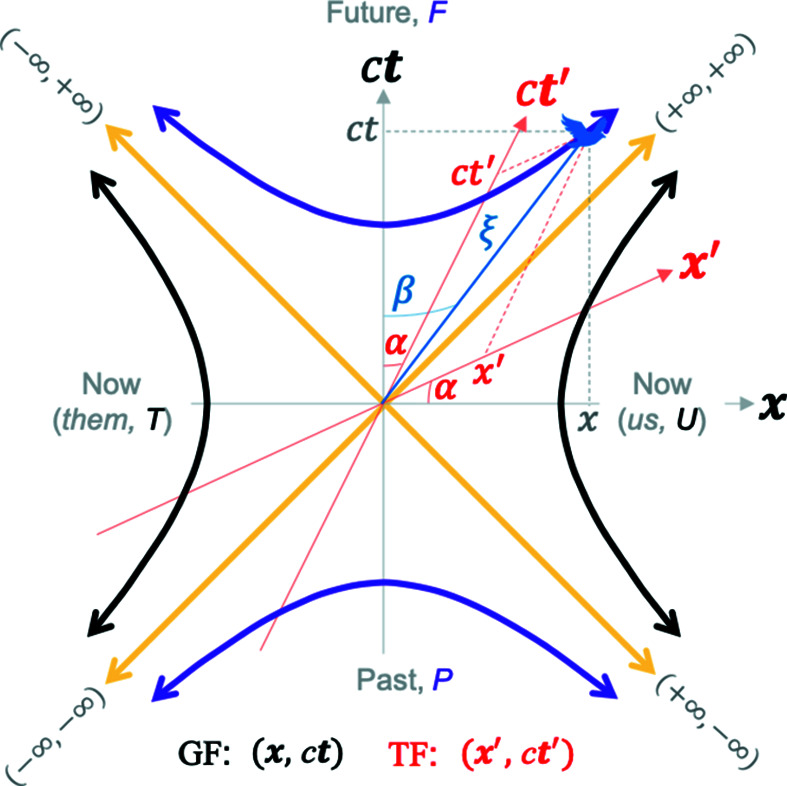
A 2D real Minkowski spacetime depicts hyperbolas given by 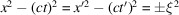 , where the purple pair of hyperbolas correspond to 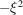 (time-like events) and the black pair of hyperbolas to 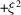 (space-like events). An arbitrary time-like event is shown by a blue line from the origin to the event (the blue bird), and the projection of its coordinates 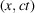 = 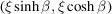 and 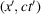 = 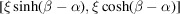 is depicted by broken lines on to the ground (GF, black) and the train (TF, red) frames. The diagonal yellow lines are the light lines given by 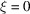 ; their poles 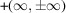 and 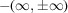 are indicated. The four hyperbola branches are labeled *F*, *P*, *U* and *T*. See the *Mathematica* script in the *Mathematica* notebook in the supporting information to generate this plot.

In contrast, the geometry of special relativity is hyperbolic as described elegantly by Dray (2012 ▸). Fig. 1 ▸ schematically defines the three inertial frames of relevance in this work, which for pedagogical purposes we label as the ground frame (GF), the train frame (TF) and the bird frame (BF). The TF and BF move at a velocity of *v* and *u* relative to the GF, respectively. Two inertial observers, one in the GF and another in the TF (depicted by the silhouette of girls shown on the ground and on the moving train, respectively, in Fig. 1 ▸), are observing an event (the bird flying) whose coordinates are measured in the GF as (*x, ct*), and in the TF as (*x′, ct′*), where *c* is the speed of light in vacuum. The hyperbolic angles 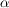 and 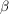 can be defined by the relative frame velocities, given by 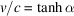 and 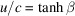 . A geometric construction illustrating the significance of the hyperbolic angles is shown in Fig. 2 ▸. The frame co-moving with the event (*i.e.* flying with the bird, or the so-called bird frame, BF, in Fig. 1 ▸) is typically called the proper frame, or the wristwatch frame.

In 2D conventional relativistic spacetime spanned by unit vectors **x** (space axis) and **t** (time axis), 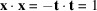 (note the minus sign). In other words, if two inertial observers, GF and TF, moving at a relative velocity of *v* to the GF (Fig. 1 ▸), observe the same event (bird) and record its coordinates as 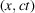 and 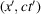 , respectively, then,

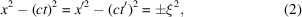

where 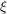 is called the spacetime length, 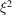 is called the spacetime interval, 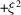 corresponds to spacetime directions from the origin along which space-like events occur (the *them*, *T*, and *us*, *U*, hyperbola branches shown with black lines in Fig. 2 ▸ represent such events with a constant spacetime length) and 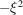 to directions from the origin where the time-like events occur (the *future*, *F*, and *past*, *P*, hyperbola branches shown as purple lines in Fig. 2 ▸ represent such events with a constant spacetime length). Equation (2) thus describes hyperbola branches in the **x** − *c* **t** plane for a fixed 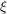 . In flat spacetime, 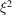 is invariant across all inertial frames, *i.e.* independent of their relative velocity *v*. In 2D, Lorentz transformation relates the coordinates of an event (the bird) between a GF, 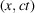 , and a TF, 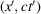 , moving along the +*x* axis with a speed of *v*, as follows:

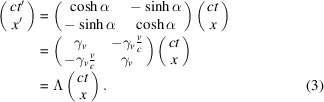

In equation (3), 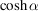 = 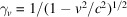 , 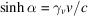 , and hence 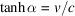 . Furthermore, 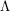 , a 2 × 2 matrix with a determinant of 1, represents the Lorentz *boost*. It is also readily confirmed that equations (2) and (3) are consistent.

In an effort to place space and time on an equal footing, Poincaré (1906 ▸) and later Minkowski (1910 ▸) defined an imaginary time ( 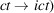 such that a spacetime interval is defined now as 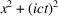 . Clearly, 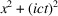 = 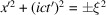 looks like a Euclidean norm and is identical to equation (2). However, Misner, Thorne and Wheeler bid ‘farewell to *ict*’ in their classic book *Gravitation* (Misner *et al.*, 1973 ▸), providing several reasons for doing so: suppression of the underlying metric structure [( 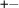 ) in the 2D spacetime], hiding the distinction between covariant and contravariant quantities, hiding the interlocking causal structure imposed by the light cones, and not being generalizable to curved spacetime. Pedagogically, an imaginary time is somewhat non-intuitive.

Several authors in the past have proposed geometric constructions [see Guillaume (1918 ▸), Mirimanoff (1921 ▸), and Gruner & Sauter (1921 ▸), Gruner (1921 ▸) for its historical roots] that avoid imaginary time, and instead use real space and time coordinates. One such construction by Enrique Loedel Palumbo in 1948 (Loedel, 1948 ▸) was rediscovered independently by Henri Amar in 1955 (Amar, 1955 ▸), and later re-rediscovered independently by Robert W. Brehme in 1961 (Brehme, 1962 ▸). This construction (referred to here as the LAB construction) makes the choice to draw the axes 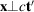 and 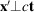 , a construction we will revisit next.

## 2. Blended spacetime coordinates, (*x, ct*′) and (*x*′, *ct*) yield a Euclidean geometry   

Rearranging terms in (3), one arrives at the following:

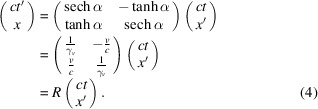




This represents a Lorentz transformation between 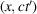 and 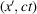 coordinates. Together, they are referred to here as a pair of blended coordinates composing a *blended spacetime*. These blended coordinates can be thought of as two inertial observers adopting each other’s clock readings, while each retains their original inertial spatial coordinates. (Equivalently, they can adopt each other’s spatial coordinates while retaining their own clocks.) This can trivially be performed in a passive manner, post-measurement, assuming each observer knows special relativity and the two have an agreed-upon origin. By redefining 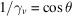 , 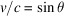 and 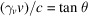 , we can rewrite equation (4) as follows:

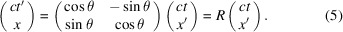

Further, by rearranging equation (2), we get

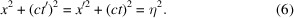

If we define 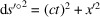 and 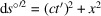 as the spacetime intervals in the blended coordinates, we gather from equation (6) that 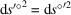 . These intervals describe the Euclidean interval between the event and the origin in the blended spacetime frames 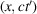 and 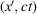 , generated by the blending of the GF and TF observers in Fig. 1 ▸. This looks like a Euclidean measure. The Euclidean interval 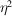 is however not an invariant across different inertial frames in the MS; it is a function of both *v* and *u*, as derived next.

If we write 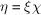 , then equation (6) motivates us to define blended Euclidean coordinates as follows:

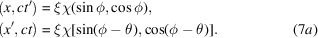

Here, the angle definitions are: 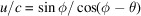 (for events along time-like directions in MS), 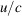 = 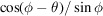 (for events along space-like directions in MS) and 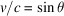 . In other words,

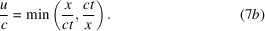

Note in particular that these definitions ensure that 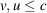 .

To find an expression for 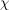 as a function of the Euclidean angles, we substitute the coordinates of equation (7*a*)[Disp-formula fd7a] into equation (2) for events observed from the GF, namely, 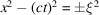 . One finds that 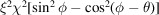 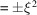 ; here the positive sign is for space-like events and the negative sign for time-like events. Upon simplification, this leads to 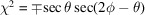 , where the negative sign is for space-like events and the positive sign for time-like events. Alternatively, one could substitute the hyperbolic coordinates of a general event from Fig. 2 ▸ into equation (6) to show that 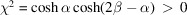 , since a cosh function is always positive. One could therefore equivalently write 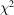 = 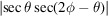 (in order to ensure that it stays positive for all Euclidean angles) and hence

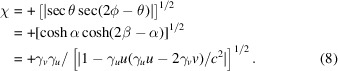

Here the positive root is chosen without a loss of generality, and 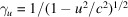 .

**Figure 3:**
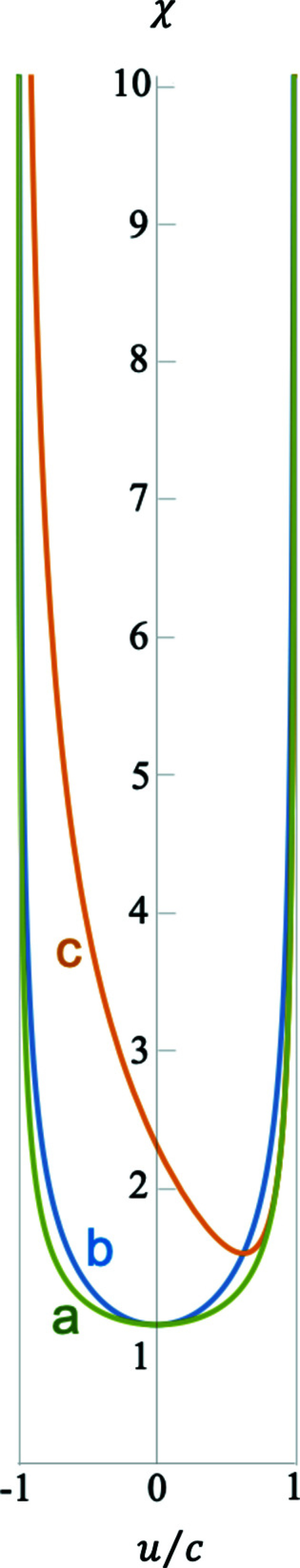
Plots of the renormalization factor 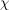 from equation (8) as a function of 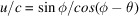 for (*a*) 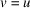 , 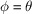 , (*b*) 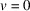 , 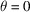 , (*c*) 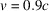 . The light lines correspond to the vertical asymptotes at 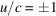 . See the *Mathematica* script in the *Mathematica* notebook in the supporting information to generate this plot.

In a similar fashion, substituting equation (7*a*)[Disp-formula fd7a] into equation (2) for events observed from the TF, 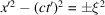 , we get the same expression for 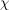 as noted above. The term 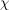 is called the *renormalization factor*, and is plotted in Fig. 3 ▸ as a function of 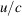 for three different values of 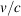 , namely, 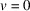 , 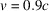 and 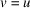 . These three cases will be explored further in the following sections. The light lines are the vertical asymptotes at 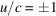 where the 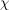 diverges (*i.e.* 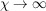 ).

**Figure 4:**
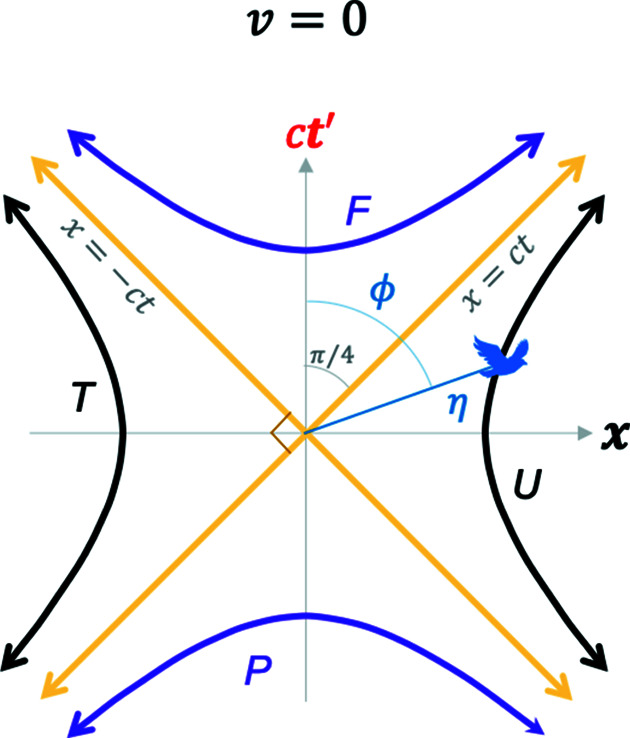
A plot of the 2D Euclidean blended spacetime coordinates in equation (7*a*)[Disp-formula fd7a] with equation (8) substituted in it, for 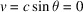 . The hyperbolas in Fig. 2 ▸ are recovered but the angles are now Euclidean. For this case, the **x **and 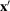 coordinates are coincident (horizontal axis), and the *c* **t** and 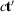 axes are coincident (vertical axis), and 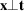 . See the *Mathematica* script in the *Mathematica* notebook in the supporting information to generate this plot.

With the Euclidean coordinates in equations (7*a*)[Disp-formula fd7a] plus (8) in hand, we are ready to replot the MS in Fig. 2 ▸ in terms of the blended and the RBS coordinates. Fig. 4 ▸ plots the coordinates of equation (7*a*)[Disp-formula fd7a] [along with equation (8)] for the special case of 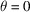 . This is the case of a stationary train in Fig. 1 ▸, with 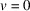 . Strikingly, one can capture all the four hyperbolas in Fig. 2 ▸ including the time-like and space-like events by varying 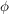 (bird flying at varying speeds, *u*). When 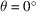 , the plot reproduces the hyperbolas and the light lines shown in Fig. 2 ▸ with the **x** and 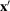 coordinates coincident (horizontal axis), 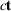 and 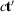 coincident (vertical axis) and 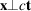 . This mathematical exercise is important since it shows that the hyperbolas in the MS can be captured equally well with Euclidean functions and angles in Fig. 4 ▸, instead of hyperbolic functions and angles as in Fig. 2 ▸.

**Figure 5:**
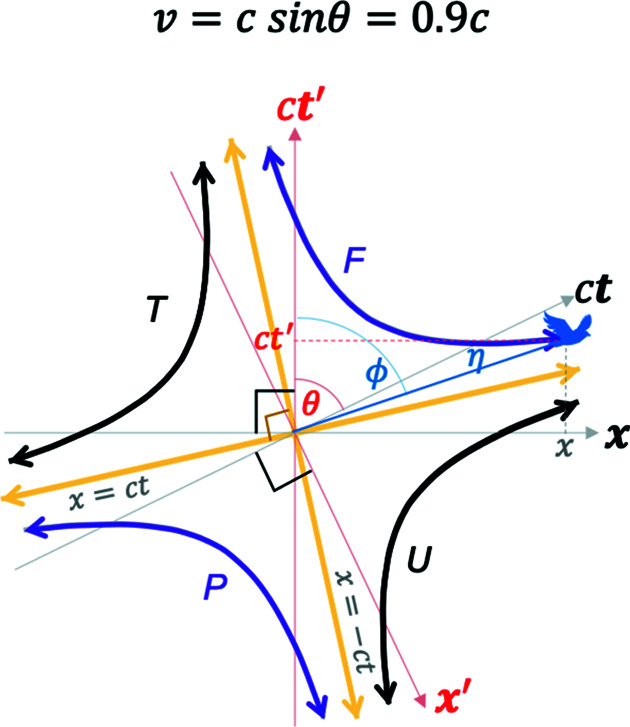
A plot of the 2D Euclidean blended spacetime coordinates from equation (7*a*)[Disp-formula fd7a] with equation (8) for 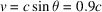 . The two light lines are oriented at the angles of 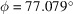 and 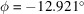 . See the *Mathematica* script in the *Mathematica* notebook in the supporting information to generate this plot.

However, when 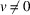 as shown in Fig. 5 ▸, the hyperbolas are rotated by a Euclidean rotation angle 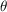 which captures the Lorentz boost [equation (5)] between the two pairs of blended coordinates. The light lines given by 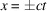 result in the condition 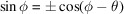 , which, for example for 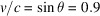 , yields the orientations of the two light lines as 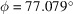 and 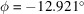 as shown in Fig. 5 ▸.

## 3. Renormalized blended spacetime (RBS) coordinates   

Rearranging equation (7*a*)[Disp-formula fd7a], it is clear that

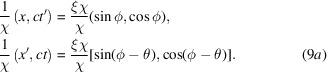




Note that we are intentionally not ‘canceling out’ the 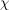 terms on the right-hand side of equation (9*a*)[Disp-formula fd9a], since 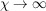 when 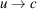 . In that special case, we should consider the limit as follows:

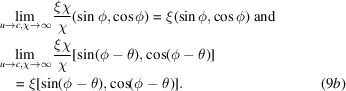




If we define the renormalized coordinates as follows:

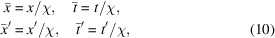

then, the RBS coordinates can be rewritten as

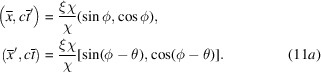




Again, in the limit of 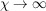 when 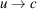 , one has to take the limits on the right-hand side using L’Hôpital’s rule, 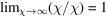 , leading to the following:

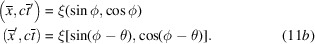

The Lorentz transformation in equation (4) can now be rewritten in the RBS coordinates as

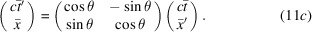

Equation (6) can be rewritten as an RBS invariant as

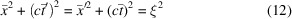

where we take the limit 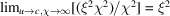 on the right-hand side. This provides the equation of a circle in the RBS coordinates. This construction is equivalent to the LAB construction (Loedel, 1948 ▸; Amar, 1955 ▸; Brehme, 1962 ▸) where the choice made to draw the axes 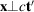 and 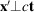 is implicit in the Euclidean coordinate choice in equation (7*a*)[Disp-formula fd7a]. Consider next, four special cases of the RBS coordinates, namely 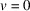 , 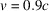 , 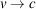 and 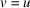 .

**Figure 6:**
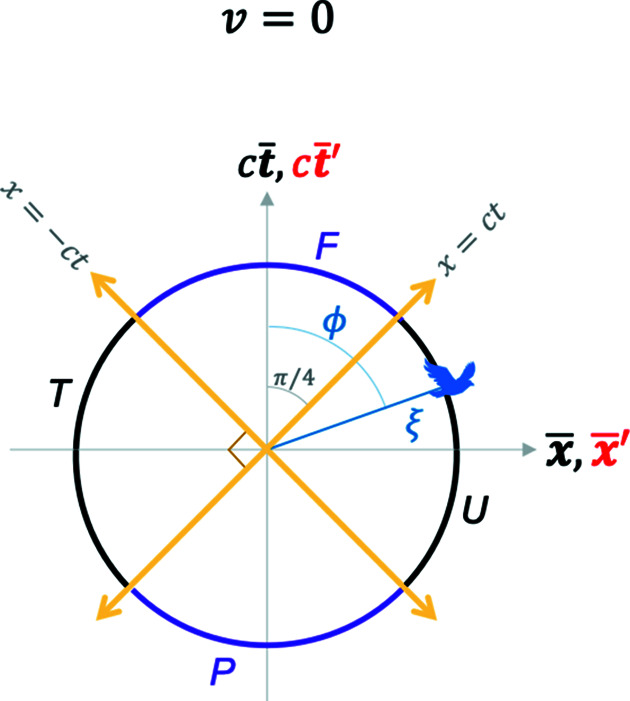
A special case of the blended and the RBS coordinates, equation (11*b*)[Disp-formula fd11b], where 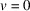 . RBS coordinates, given in equation (11*b*)[Disp-formula fd11b], transform the blended coordinates plot in Fig. 4 ▸ into a circle of radius 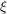 , where the four hyperbola branches in Fig. 4 ▸ become four arc segments of the circle here. Purple (black) arc segments represent time-like (space-like) events. Light lines are shown by yellow lines. See the *Mathematica* script in the *Mathematica* notebook in the supporting information to generate this plot.

*Case I*, 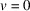 ( 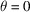 ). Here, the GF and the TF observers are coincident; this could be considered as the limit where the GF observer is self-blending. Upon renormalization by 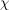 according to equation (10), the four hyperbola branches depicted in Fig. 4 ▸ transform into four arc segments of a circle as shown in Fig. 6 ▸, two of them time-like [purple segments, where 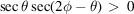 ], and the other two space-like [black segments, where 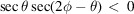 ]. This is essentially the case of a renormalized Minkowski spacetime, or RMS. Blending is essentially missing here; hence it is one of the simplest cases of ‘Euclideanizing’ MS.

The RBS coordinates also possess RBS light lines as 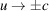 . To see this, consider that the light lines are defined in the MS by 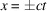 . When 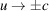 , 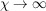 from Fig. 3 ▸. From equation (11*b*)[Disp-formula fd11b], 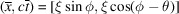 ; hence the light lines correspond to the condition 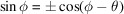 . This equality has a solution for 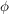 given any value of 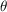 . For example, when 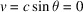 , the two RBS light lines are at angles of 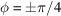 as shown in Fig. 6 ▸. The corresponding coordinates for the light lines in the MS are therefore 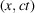 = 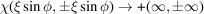 or 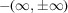 , which is consistent with the four infinity limits of the light lines in the hyperbolic construction in Fig. 2 ▸. Conversely, starting from the 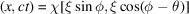 coordinates in the MS and renormalizing with 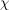 as shown in equation (9*a*)[Disp-formula fd9a], one encounters a 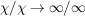 term as 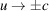 . However, there is a well defined limit of 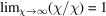 ; in this limit the RBS coordinates are 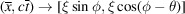 . Furthermore, as 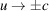 , 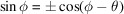 ; hence 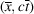 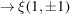 , which are the light lines shown in Fig. 6 ▸. Thus, the light lines in the RBS coordinates (Fig. 6 ▸) map to the 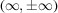 or 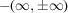 limits of the light lines in the MS coordinates (Fig. 2 ▸). We will more formally discuss these mappings in the next section.

A remarkable consequence of formulating this problem with the Euclidean angle 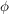 is that it can be continuously varied from 0 to 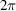 around a circle without violating any relativistic physics. This means that one can smoothly ‘rotate across’ the RBS light lines in Fig. 6 ▸ which is not possible with the hyperbolic angle, 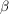 in Fig. 2 ▸. This is because in the span that 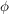 varies from 0 to 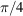 , 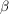 varies from 0 to 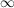 , both of which correspond to approaching the light line. Note that 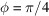 results in a well defined limit of 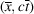 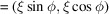 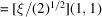 ; this point lies on the light line in Fig. 6 ▸ just as expected, the same limit that was obtained earlier when 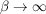 in Fig. 2 ▸. Now consider what happens when 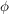 changes by an infinitesimal amount, 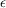 , from a value of 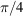 , which is a deviation from the RBS light line in either direction, *i.e.* 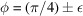 . Now, 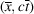 = 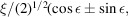 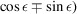 . As 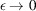 in a continuous manner, 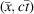 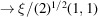 , namely one mathematically approaches the light line smoothly as expected. Thus, the mathematical crossing across the RBS light line by varying 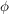 is smooth and continuous. This is a big departure from the hyperbolic construction of spacetime in Fig. 2 ▸, where one is unable to mathematically ‘cross’ the MS light lines by boosting an event frame, and hence has to ‘stay put’ in one of the four hyperbolic branches for a finite spacetime length, 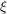 . We will have more to say about the formal mapping between the MS and RBS spaces in the next section.

**Figure 7:**
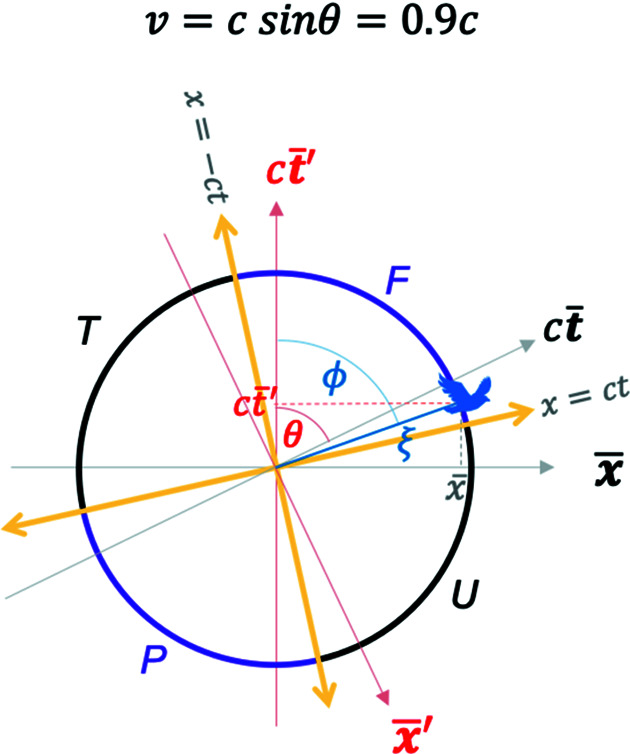
Renormalized blended spacetime (RBS) coordinates that turn the four hyperbolas (*F, P, U* and *T*) in Fig. 5 ▸ to arcs of a circle. An arbitrary event (a bird) and its RBS coordinates are depicted. See the *Mathematica* script in the *Mathematica* notebook in the supporting information to generate this plot.

*Case II*, 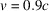 . In this case, the hyperbola branches in the blended coordinates in Fig. 5 ▸ transform into arcs of a circle in the RBS coordinates of equation (11*b*)[Disp-formula fd11b]. This is shown in Fig. 7 ▸. The orientation of the RBS light lines is found by exploring the limit of 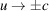 , 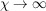 (see Fig. 3 ▸). From equation (11*b*)[Disp-formula fd11b], 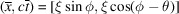 ; hence the RBS light lines correspond to 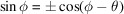 . When 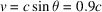 , the two RBS light lines are at angles of 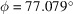 and 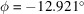 , respectively, as shown in Fig. 7 ▸. Interestingly, the RBS light lines rotate in the Euclidean plane as *v* varies. This is explored further next.

**Figure 8:**
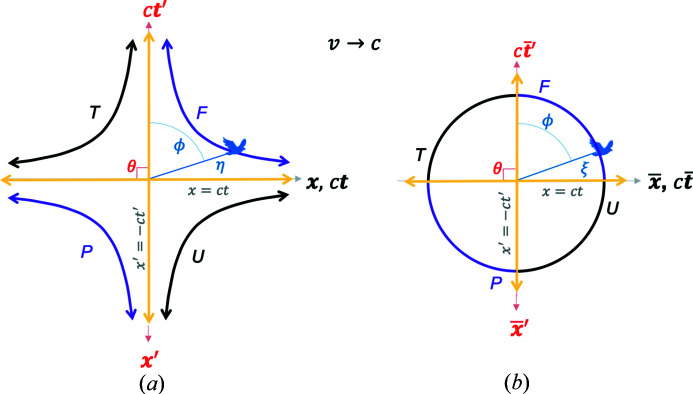
A special case of the blended and the RBS coordinates, where 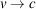 . (*a*) A plot of the blended coordinates given in equations (7*a*)[Disp-formula fd7a] and (8). (*b*) RBS coordinates, given in equation (11*b*)[Disp-formula fd11b], transform (*a*) into a circle of radius 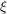 , where the four hyperbola branches in (*a*) become four arc segments of the circle. Purple (black) hyperbola branches and arc segments represent directions from the origin where time-like (space-like) events occur for a fixed 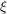 . Blended and RBS light lines are shown by yellow lines. See the *Mathematica* script in the *Mathematica* notebook in the supporting information to generate these plots.

*Case III*, 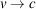 . As 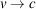 , the angle 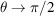 . This is a case of blending between the GF and the TF where the latter is moving at 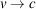 . The resulting blended and RBS frame plots are shown in Fig. 8 ▸. The light lines for this case can be found by setting 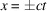 and 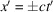 . From the coordinates in equation (7*a*)[Disp-formula fd7a] and in the limit of 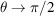 , one can therefore rewrite these relations as 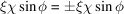 and 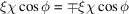 . These relations imply that the RBS light lines correspond to 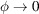 and 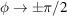 as shown. As in the previous case, one can show that for 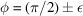 or 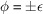 , the RBS coordinates smoothly approach the RBS light lines as 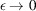 .

*Case IV*, 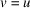 . Here the TF and BF merge into each other, *i.e.* the case of a proper frame. This can also be deduced by noting that when 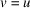 in Fig. 5 ▸, 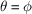 , and the coordinate 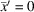 , which corresponds to the set of events on the 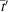 axis in Fig. 2 ▸; by definition, those events are occurring in the proper frame.

**Figure 9:**
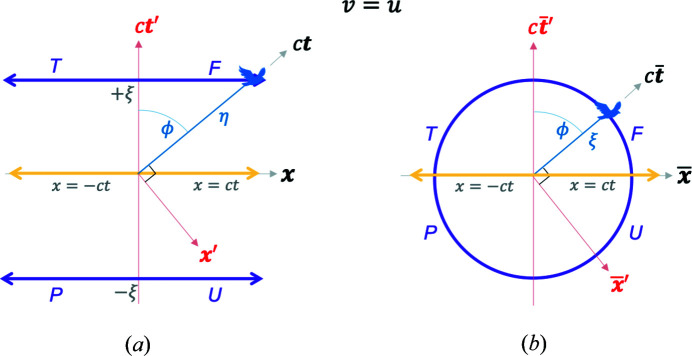
A special case of the blended and the RBS coordinates, where 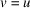 , and hence 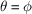 . (*a*) A plot of the blended coordinates given in equation (7*a*)[Disp-formula fd7a] with equation (8) substituted in. (*b*) RBS coordinates, given in equation (11*b*)[Disp-formula fd11b], transform (*a*) into a circle of radius 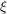 , where the four hyperbola branches in (*a*) become four arc segments of the circle. Remarkably, all the arc segments now represent directions from the origin along which time-like events occur. Blended and RBS light lines are shown by the horizontal yellow line. See the *Mathematica* script in the *Mathematica* notebook in the supporting information to generate this plot.

When the GF and the BF are blended without renormalization, one gets the blended spacetime plot in Fig. 9 ▸(*a*). While in the other cases (I–III) discussed in the text, *v* (and hence TF) could have been thought of as fixed while *u* varied, in the case of 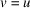 , the TF is moving along with the event. It is an unusual (but a mathematically allowed) case of a coordinate system 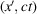 that is moving with the event frame in MS. In other words, let us say the GF girl observes an event 1 with a spacetime length of 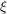 in the MS frame. This event becomes the ‘bird’. Now she blends her coordinates with the proper coordinates in the BF of event 1. If she now observes a different event 2 with a spacetime length of 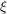 but a different boost than for event 1, she again repeats the process by blending with the proper frame of event 2. The GF is thus directly blending with the proper frame of any event she observes at a spacetime length of 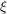 from the origin and with varying boosts.

In this special case, the hyperbola branches in the conventional spacetime in Fig. 2 ▸ flatten into straight horizontal lines at 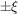 . This is understood mathematically as follows. The plot of 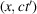 = 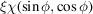 , where 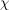 = 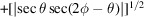 can be simplified for this case of 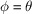 to 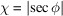 . Hence, 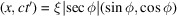 . The reason for the ‘flattening’ of the 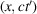 plots is due to the 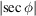 function, which diverges (*i.e.* 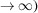 at 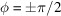 . Thus, the coordinate 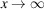 diverges, while 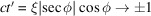 . This defines the two purple horizontal lines shown in Fig. 9 ▸(*a*).

When renormalized by 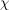 according to equation (11*a*)[Disp-formula fd11a], one gets a circle of radius 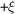 as shown in Fig. 9 ▸(*b*). Remarkably, all the events in both Figs. 9 ▸(*a*) and 9 ▸(*b*) are along time-like directions! This is seen by starting with the blended coordinates in equation (7*a*)[Disp-formula fd7a] when 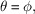 namely, 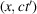 = 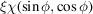 and 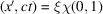 , where from equation (8), 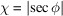 . By substituting into equation (2), one gets 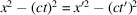 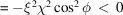 , which indicates a time-like direction.

Another unusual aspect of this case is that the two light lines merge into a single blended or RBS light line parallel to the 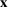 axis, as shown. The light lines are defined by 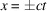 , which is equivalent to 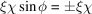 , which suggests that 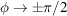 as shown. The light lines are also defined by 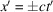 , which implies 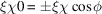 , which again yields 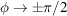 . This implies that the blended and RBS light lines coincide with the horizontal *x* axis in Fig. 9 ▸(*a*). This is perhaps the simplest and somewhat surprising RBS geometry one could imagine: a time-like circle of constant RBS interval with a single light line.

## 4. Mapping of events from the Minkowski to the RBS coordinates   

Now we formally explore the transformation and the type of mapping between the MS and the RBS coordinates. We explore two cases: MS 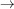 RBS (in this section) and RBS 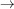 MS (in the next section). This will be used to validate that the RBS coordinates do indeed capture the relativistic physics content of the MS coordinates.

Consider the transformation from the conventional rest frame in the MS to the RBS frames as follows:

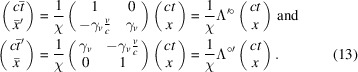




Similarly, the transformation from the moving frame, TF, to the RBS frames is as follows:

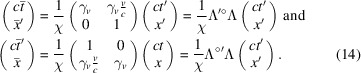




Consider now starting from a general coordinate 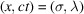 in the conventional spacetime frames in Fig. 2 ▸. How do they transform into the blended coordinates? From equation (3), 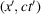 = 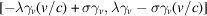 . By renormalization with 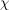 , we get the blended coordinates 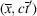 = 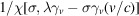 and 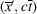 = 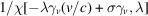 . For 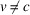 , every MS event thus has unique and well defined RBS coordinates.

**Figure 10:**
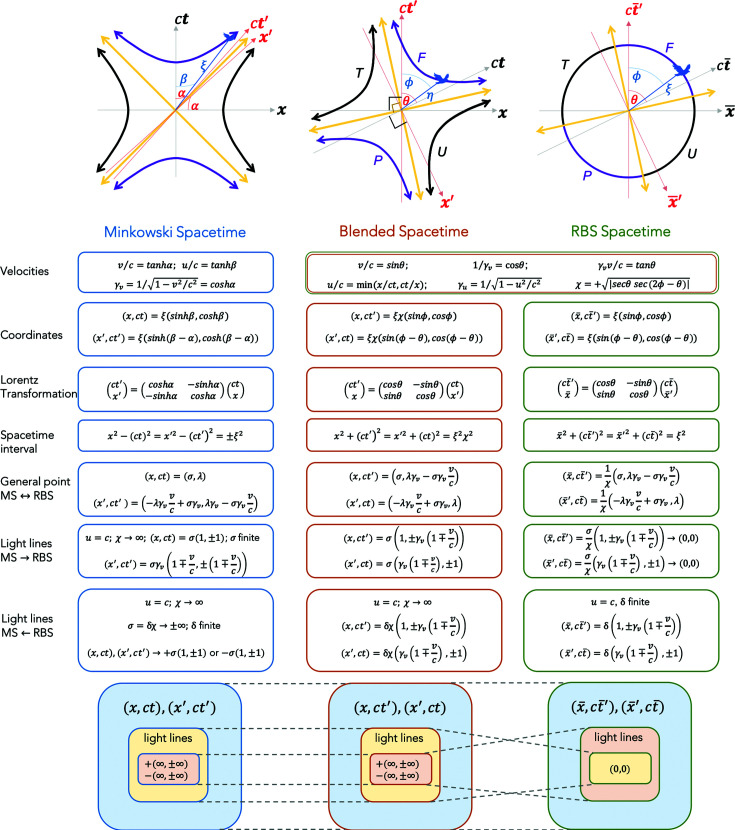
A summary of representative diagrams (top row diagrams simplified from Figs. 2 ▸, 5 ▸ and 7 ▸ for 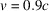 ) and the key equations mapping the Minkowski spacetime (MS), blended spacetime and the renormalized blended spacetime (RBS). The bottom row schematics indicate the mappings between the three spacetimes shown by the dashed gray lines. In addition to the MS, blended spacetime and RBS coordinates, one could also express three more coordinates: Minkowski polar ( 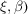 , blended polar ( 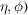 and renormalized blended polar ( 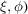 . Relations between all six of these coordinate systems can be deduced from the above information and that given in the main text.

How about the events along the light lines, 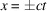 ? In this case, 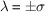 for which 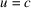 . Then, 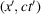 = 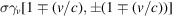 , 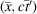 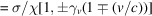 and 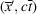 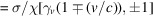 . From Fig. 3 ▸, as 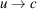 , 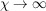 . If 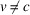 and 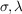 are finite, then 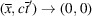 and 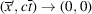 . Thus, the events with finite coordinates on the two MS light lines in Fig. 2 ▸ map to the RBS origin (such as in Figs. 6 ▸ and 7 ▸), a many-to-one mapping from the MS to the RBS. This is summarized in Fig. 10 ▸.

In linear algebra, this is expressed as follows: the kernel (or nullspace) of the transformation 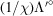 [and the transformation 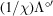 ] from the MS to the RBS coordinates is the set of all events that form the light line in the MS, namely, the lines 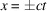 . The range of the transformation matrix 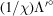 is the 2D blended vector space spanned by the column vectors of this transformation matrix, namely 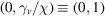 and 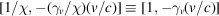 . The domain of the transformation is spanned by the column vectors of the inverse of the 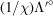 matrix.

What about the 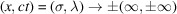 corresponding to the infinity limits of the light lines in the MS frame? This again corresponds to 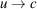 , and hence 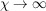 . In the next section, it is shown that in the limit of 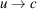 , the four infinity limits of the light lines, 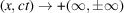 and 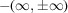 , map to finite, well defined coordinates in the RBS. These results are also summarized in Fig. 10 ▸.

## 5. Mapping of events from the RBS to the MS coordinates   

Consider a general event coordinate given by 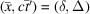 in Figs. 6 ▸ or 7 ▸ in the RBS frame. Using equations (4) and (10), one can determine the corresponding coordinates in the 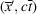 frame and in the MS frames as follows. From the definition of the normalized coordinates, it follows that 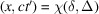 . From equation (4), it follows that 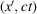 = 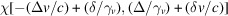 . Renormalizing for a finite 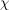 according to equation (10), one can find that 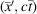 = 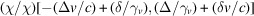 . For 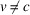 , all of these coordinates are well defined, and there is a well defined mapping from the RBS to the MS coordinates and between the two RBS frames.

How about the events along the light lines, 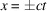 in the RBS coordinates in Figs. 6 ▸ or 7 ▸? In this case, from above, 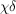 = 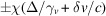 . Rearranging we get 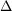 = 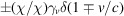 . Substituting this relation into the MS coordinates above, we get 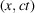 = 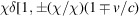 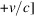 . However, light lines correspond to 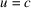 , and hence 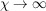 . Hence, 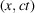 = 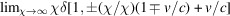 = 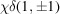 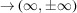 or 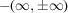 , depending on the sign of 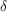 . Thus, any arbitrary event 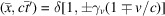 on the light lines in the RBS frame (Figs. 6 ▸ or 7 ▸) maps to one of the four infinity limits, 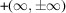 or 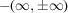 , of the light lines in the MS frame (Fig. 2 ▸) as shown in Section 4.

## 6. Summary of important results thus far leading to the RBS coordinates   

We pause to summarize the relationships between the MS, blended spacetime and the RBS. This is done through Fig. 10 ▸ where the important equations and representative diagrams are presented for each spacetime. The information content in all three frames in terms of relativistic physics is equivalent, *i.e.* all essential physics is captured in translating between these frames. For 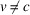 , every MS event has unique and well defined RBS coordinates. Light lines in the MS frame map to the origin in the RBS frame, while the light lines in the RBS frame map to the 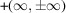 and the 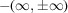 poles in the MS frame. This is an example of a transformation where points at infinity in the MS are transformed to finite Euclidean points in the RBS. Both frames have a pair of equivalent light lines that capture the same physics. Among significant qualitative differences, the MS does not allow for a mathematical ‘crossing’ of the light line through a hyperbolic Lorentz boost, while this is possible in the RBS as shown above. This can be succinctly understood as follows: in hyperbolic geometry, a ‘time-like event’ is represented by the coordinates 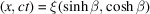 which approaches 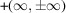 and 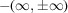 as the event frame is boosted and it approaches the light lines; hence its coordinates diverge, and the event frame can only approach the light lines asymptotically. Using the RBS transformation, these infinity limits of the MS light lines can be transformed to finite Euclidean RBS coordinates, given by 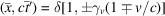 . Now the RBS light lines can be ‘approached’ and even ‘crossed’ upon boosting an event frame.

There is no contradiction in the relativistic physics between the two formalisms. For example, consider the simple case of 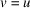 in the RBS coordinates as shown in Fig. 9 ▸. This case leads to the condition 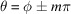 , where *m* is an integer, and 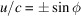 , which places no restriction on the angle 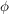 . The RBS light lines in this case are at 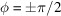 . When 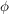 increases from zero to 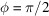 , the event frame velocity, 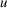 , increases from zero to 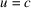 . Upon crossing the RBS light line at 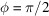 , when 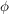 exceeds 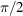 , the *u* according to equation (7*b*)[Disp-formula fd7b] slows down back from *c* and approaches a value of zero when 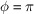 . In the range 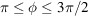 , the *u* speeds up again to equal −*c* upon approaching the light line at 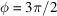 . Finally, after ‘crossing’ the RBS light line a second time, the event slows down again to zero upon reaching 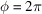 . All of this is consistent with Einstein’s postulates in flat spacetime; at no point does the speed, *u*, of the event frame exceed *c*.

Now consider the case when 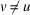 . In all these cases in Figs. 4 ▸, 5 ▸ and 6 ▸ for example, there are two light lines. Consider the specific case of 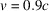 in Fig. 5 ▸ and recall that 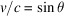 , and in accordance with equation (7*b*)[Disp-formula fd7b], 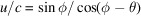 (for events along time-like directions) and 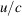 = 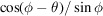 (for events along space-like directions). Starting from 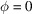 , which corresponds to 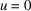 , and traveling along the *F* branch, upon reaching the first RBS light line at 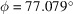 , 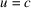 . At this stage, the event switches from being in time-like directions to space-like directions on branch *U*, and *u* starts decreasing back down from *c*. When 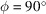 , 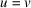 . Upon further travel along the *U* branch, when 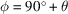 , 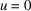 . Continuing further on the *U* branch and reaching the second RBS light line crossing at 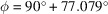 , 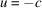 . As 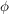 increases further, *u* decreases, while the events are now along time-like directions again on the arc *P*. At 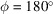 , 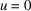 . On further travel along the arc *P*, the speed *u* increases again until it reaches 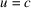 at 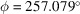 where it meets the RBS light line again. Beyond that, the events again switch to lying along space-like directions on the arc *T*. At 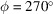 , 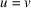 , and at 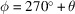 , 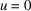 . At 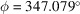 , we mathematically cross the RBS light line again, and 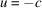 . Beyond that, the events are again back on arc *F* along time-line directions. At 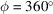 , 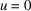 , and we are back a full circle.

## 7. Lorentz and Poincaré groups in the RBS coordinates   

Consider now a generalization to the Minkowski 4D spacetime, defined by the three contravariant space coordinates, 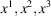 , and time coordinate, 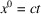 . The proper Lorentz group, 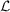 , comprises six operations within an isotropic 4D spacetime (Corson, 1953 ▸; Başkal *et al.*, 2015 ▸): three independent Euclidean rotations, 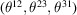 , respectively, within one of the three space planes, 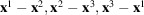 . Their coordinate transformation matrices are, respectively, given by

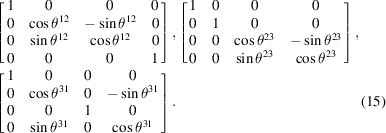




In addition, 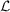 has three independent Lorentz boosts, 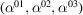 [similar to 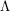 , in equation (3)], each, respectively, within one of the spacetime planes, 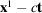 , 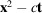 , 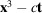 . Their coordinate transformation matrices are, respectively, given by

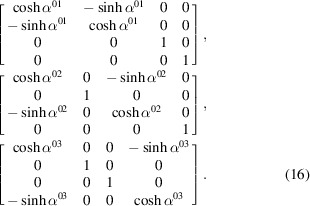




In the RBS frame, the RBS proper Lorentz group, 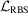 , the same spatial rotation matrices as in equation (15) are valid, except in the 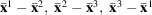 planes, respectively. However, one notices from equation (5) that the Lorentz boosts given in equation (16) can instead be written as Euclidean rotations. The three Lorentz boosts in equation (16) are now rewritten in the RBS frame as Euclidean rotations:

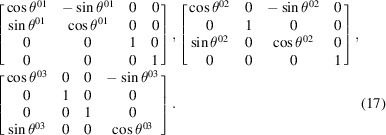




Here, 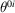 ( 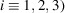 are the three Euclidean Lorentz boost angles in the 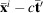 planes given by 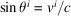 . Typically, one defines these rotations in the range 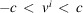 , which translates to 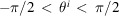 . However, one is allowed to vary 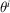 smoothly across the light lines in the RBS coordinates in the range 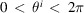 , without violating any relativistic physics; the maximum 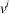 will still remain *c* as discussed in the previous section.

If now RBS spacetime inversion, 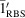 , is defined as 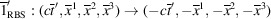 , RBS time reversal as 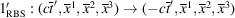 and RBS spatial inversion as 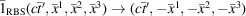 , then 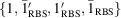 forms a group, 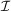 , where 1 stands for the identity matrix. Through a direct product of the proper RBS Lorentz group with the group 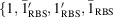 , *i.e.* 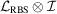 , a new group is created, called the extended RBS Lorentz group, 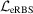 (Corson, 1953 ▸; Başkal *et al.*, 2015 ▸). [A note on notation: in crystallography, 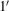 denotes time reversal; the superscript ‘prime’ has nothing to do with the ‘prime’ used to represent the train frame, TF, here in special relativity. Similarly, the overbar such as 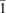 in conventional crystallography is used to denote spatial inversion; it has nothing to do with the overbar used here for renormalization as in equation (10). The coincidence is unfortunate, but the context should provide clarification: the use of prime and overbar in conjunction with symmetry elements represent time reversal and spatial inversion, respectively. If the prime and overbar are used in conjunction with spacetime coordinates, as in equation (10), they represent TF and renormalization respectively.]

How about translations? So far, we have described spacetime intervals observed from a common origin by GF and TF observers in MS or by the blended observers in RBS. A general translation would move the origin, which would, in general, rescale the spacetime interval for a given event. In an infinite space crystal with translational symmetry, there is a periodic placement of atoms. Space groups describe their global symmetry while point groups describe the local (site) symmetry at individual locations within the crystal. Similarly, one could create translational symmetry in a spacetime crystal with periodic placement of events, where the global symmetry is described by the Poincaré space groups and the local (site) symmetry is described by the Lorentz point groups. In such a case, a single observer (conventional or blended) at a selected origin would observe all of these infinite series of periodic events in the manner described so far with Lorentz groups. Just as in space crystals, translations would also create a periodic set of observers (conventional or blended) related by translational symmetry, each observing an identical environment of events around them. The translational symmetry of spacetime captured by Poincaré groups is discussed next.

The proper Poincaré group, 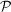 , in 4D MS coordinates consists of the proper Lorentz group, 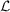 , combined with four translations, namely, 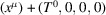 , 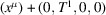 , 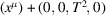 and 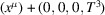 , where 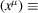 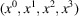 and 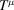 are the translations along the coordinates 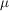 that vary from 0 to 3 (Corson, 1953 ▸; Başkal *et al.*, 2015 ▸). If, in addition, improper transformations are included, namely spatial inversion, 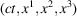 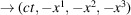 , and time reversal, 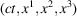 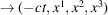 , then one forms an extended Poincaré group, 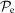 (Corson, 1953 ▸; Başkal *et al.*, 2015 ▸).

The proper RBS Poincaré group, 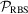 , in 4D coordinates is similarly defined as the proper RBS Lorentz group, 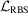 , plus four translations, namely 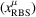 + 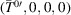 , 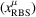 + 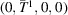 , 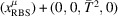 and 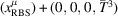 , where 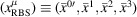 and 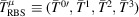 are the translations along the RBS coordinates, 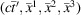 , respectively. If these translations are included in the extended RBS Lorentz group, 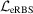 , one gets an extended RBS Poincaré group.

## 8. 2D RBS point groups   

A striking mathematical consequence of this formulation is that the RBS Lorentz and Poincaré groups can now be mapped to the Euclidean point and space groups for space crystals, respectively; the latter are all fully listed (Aroyo *et al.*, 2011 ▸; Brown *et al.*, 1978 ▸; Palistrant, 2012 ▸). Space crystals in various dimensions can be classified into point and space groups: 17 space and ten point groups in 2D; 230 space and 32 point groups in 3D; 4895 space and 271 point groups in 4D, and so on (Aroyo *et al.*, 2011 ▸; Brown *et al.*, 1978 ▸; Palistrant, 2012 ▸). In contrast, to the best of my knowledge, only a handful of relativistic crystal groups (in 2D) have been listed so far (Janner & Ascher, 1969 ▸).

**Figure 11:**
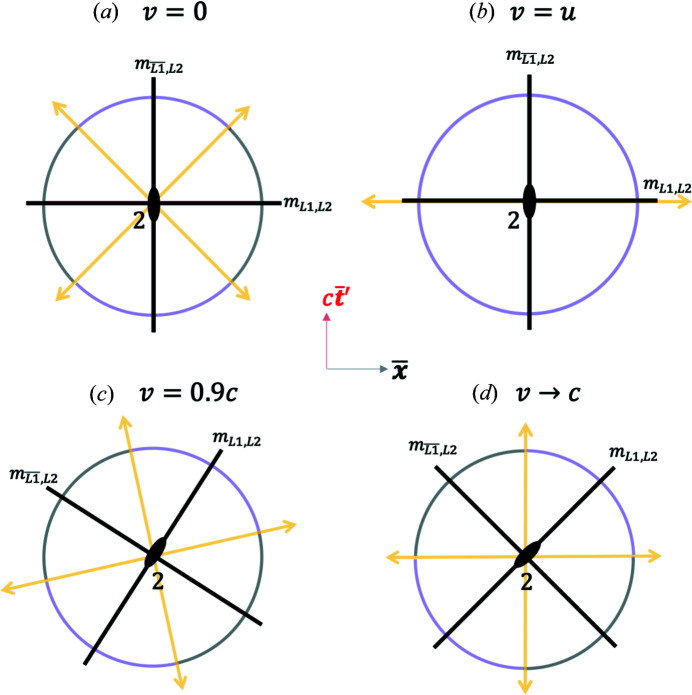
The RBS plots from Figs. 6 ▸, 9 ▸(*b*), 7 ▸, 8 ▸(*b*) reproduced here in a lighter hue as panels (*a*), (*b*), (*c*) and (*d*), respectively. The symmetry elements of the extended RBS point group ***mm*2** are overlaid on each diagram indicating the twofold rotation at the center (black oval), and the two mirrors (black lines).

Let us first begin with 2D point groups, so called because all the symmetry elements of the group must leave the coordinates of at least one point in the object or spacetime unchanged (invariant). In the discussion below, we will work from the RBS plots in Figs. 6 ▸, 7 ▸, 8 ▸(*b*) and 9 ▸(*b*) in order to identify the relevant symmetry groups. We notice in these figures two features that are important to consider: light lines, and events at a fixed RBS spacetime length of 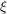 along space- versus time-like directions from the origin, represented by black and purple arc segments, respectively. We consider black versus purple line segments to be related by a color symmetry as discussed further later. Consider the following cases:

*Colorless symmetry including all the features of the RBS diagrams*. If one pays attention to the RBS light lines and the distinction between space- versus time-like directions, one notices a point-group symmetry of ***mm*2** in the RBS diagrams of Figs. 6 ▸, 7 ▸, 8 ▸(*b*) and 9 ▸(*b*). (Group labels are shown in bold font, while the elements of the group are shown in roman; the term ‘colorless’ recognizes the presence of black versus purple arc segments, but does not introduce a new symmetry element to switch between the two, as is done later on.) This is depicted in Fig. 11 ▸. The complete point group for Figs. 6 ▸, 7 ▸, 8 ▸(*b*), 9 ▸(*b*) is given as ***mm*2** 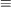 {1, 2, 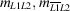 }. The element 1 represents identity. The element 2 represents a twofold rotation (*i.e.* a rotation of 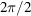 ) in the 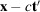 plane. Note that such a proper rotation transformation as the twofold does not exist in the original MS construction of Fig. 2 ▸. This is thus a hidden symmetry revealed in the RBS construction. The two mirrors 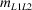 and 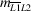 bis­ect the four quadrants formed by the RBS light lines, labeled by the subscripts *L*1 and *L*2 here. [Note that there is only one RBS light line in Fig. 9 ▸(*b*), hence one of the mirrors is parallel to the light lines, and the other perpendicular to it.]

Four other subgroups of this symmetry group are also valid groups describing the 2D RBS, namely, point groups 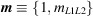 or 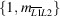 , 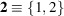 and trivially 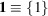 . Thus overall, there exist five 2D RBS colorless point groups: ***mm*2**, ***m*** (two possibilities), **2** and **1**. Note that, for Figs. 6 ▸ and 9 ▸(*b*), one of the mirrors is equivalent to the RBS space inversion in 2D (previously labeled 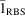 ). Similarly, the other mirror is equivalent to the RBS time reversal mentioned earlier ( 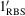 ). Finally, the twofold is equivalent to the RBS spacetime reversal, 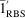 , mentioned earlier. Hence, one could alternatively represent the ***mm*2** group for the cases of Figs. 6 ▸ and 9 ▸(*b*) as 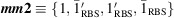 . These groups therefore represent extended RBS Lorentz groups, 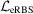 .

*Color symmetry including all the features of the RBS diagrams*. An antisymmetry such as time reversal, 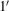 , will switch between two time-states, 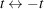 (Padmanabhan *et al.*, 2020 ▸). These two states can be associated with two colors, say black and purple, and thus 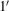 switches between black and purple colors representing the two time-states. Similarly, note that time-like and space-like events are distinguished by the parameter 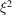 which switches sign from negative (time-like) to positive (space-like). If we introduce a new antisymmetry operation, 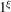 :

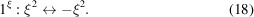

This operation thus switches the ‘color’ between time-like (purple) and space-like (black) events. One could consider implementing this operation as follows: 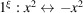 and 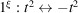 . An alternative way to perform this operation is 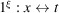 . In either case, note that neither of these operations are elements of the 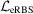 . Also note that 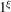 is a self-inverse (*i.e.* 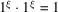 ), commutes with all the elements of the 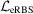 point groups mentioned earlier for the case of colorless symmetry groups which includes all the features of the RBS diagrams, and is not already an element of those groups. These are requirements for an operation to be an antisymmetry with respect to a group (Padmanabhan *et al.*, 2020 ▸).

By performing the direct product 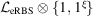 , one can generate gray RBS symmetry groups that explicitly contain 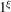 . [The ‘gray’ is supposed to reflect a mixture of black and white (here purple is chosen instead of white) because of the explicit presence of 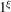 which switches between the two colors.] Its subgroups which do not explicitly contain 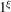 are then called the two-color RBS symmetry groups. From Figs. 6 ▸, 9 ▸(*b*), 7 ▸, 8 ▸(*b*), we can conclude that 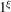 is not explicitly present, *i.e.* swapping time- and space-like events will change the diagrams, hence it is not a symmetry element of the group. Hence gray RBS groups are excluded.

**Figure 12:**
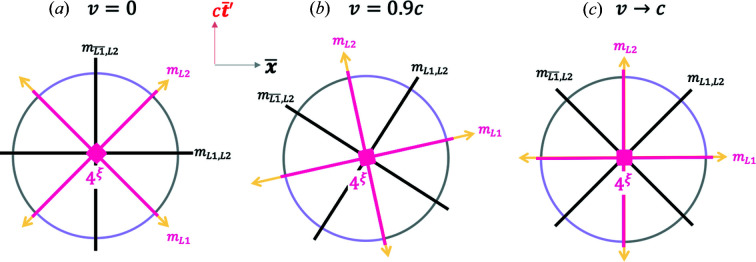
The 2D RBS plots from Figs. 6 ▸, 7 ▸, 8 ▸(*b*) reproduced here in a lighter hue as panels (*a*), (*b*) and (*c*), respectively. The symmetry elements of the extended two-colored RBS point group 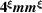 are overlaid on each diagram indicating the 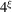 rotation axis at the center (red diamond), the two colorless mirrors (black lines) and the two-colored mirrors (red lines).

Next, we consider two-color RBS groups in analogy with two-color magnetic point groups (Litvin, 2001 ▸). Figs. 6 ▸, 9 ▸(*b*), 7 ▸, 8 ▸(*b*) exhibit the symmetry group 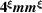 . This is shown in Fig. 12 ▸. The group elements are 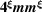 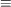 { 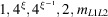 , 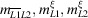 }. The elements 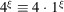 and 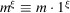 . The 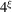 and 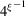 represent Euclidean fourfold rotations by an angle of 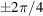 , respectively, followed by 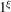 . The colored mirrors 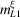 and 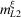 in Figs. 6 ▸, 7 ▸ and 8 ▸(*b*) are collinear with the two light lines in each figure. The uncolored mirrors 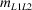 and 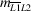 bis­ect the quadrants formed by the light lines. The subgroups of 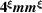 such as 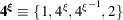 , 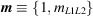 or 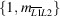 and 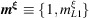 or 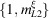 are also allowed symmetry groups for this case. In the case of Fig. 9 ▸(*b*), there are no colored symmetry elements since all events are along time-like directions.

*Colorless symmetry ignoring the RBS light lines and the distinction between time- versus space-like events*. In such a case, the symmetry group is a Curie group 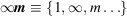 and its subgroup 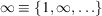 in 2D. The element 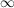 represents an infinitesimal Euclidean rotation angle of 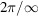 in the 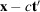 plane. The element *m* represents a vertical mirror in the plane. There are infinitely many such rotation and mirror elements in these groups, hence the ‘…’ in the group.

## 9. 3D and 4D RBS point groups   

**Figure 13:**
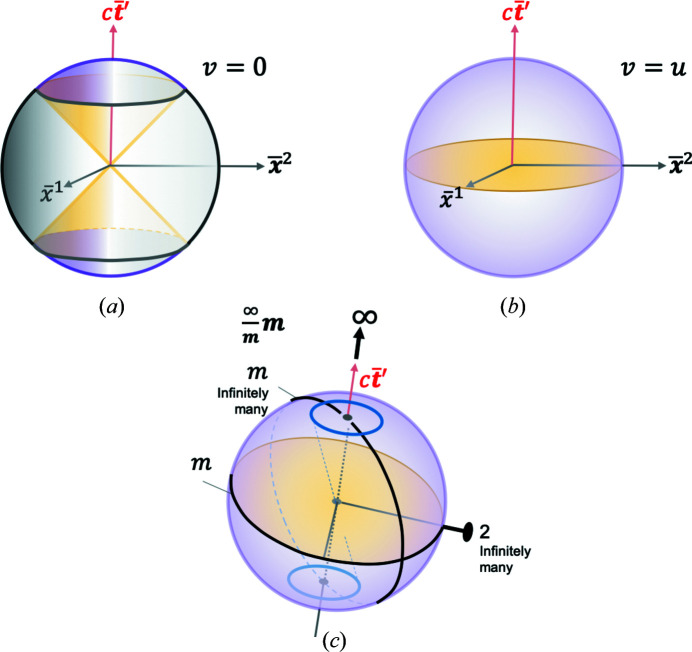
Isotropic 3D RBS coordinates depicted for (*a*) 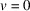 and (*b*) 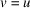 , similar to the 2D RBS in Figs. 6 ▸ and 9 ▸(*b*), respectively. The gray versus purple sphere surfaces indicate space-like versus time-like events, respectively. The yellow light cones are depicted in (*a*), while the light plane is depicted in (*b*) as the equatorial plane. Panel (*c*) depicts one 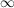 -fold rotation axis, one (of infinitely many) twofold rotation axis, one horizontal mirror and one (of infinitely many) vertical mirror. The 3D Curie point group for both (*a*) and (*b*) is 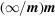 .

3D RBS would have the coordinates of 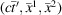 , while 4D RBS would have the coordinates of 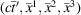 . Fig. 13 ▸ depicts 3D RBS for two cases for (*a*) 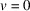 and (*b*) 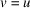 , similar to the 2D RBS in Figs. 6 ▸ and 9 ▸(*b*), respectively. The Curie group is 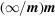 for both cases. In both cases, there is one 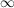 -fold axis and horizontal mirror (*m* in the denominator) in the equatorial plane perpendicular to the 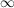 -fold axis as shown in Fig. 13 ▸(*c*). There are infinitely many vertical mirrors (*m* in the numerator), one of them is depicted in panel (*c*), and an infinite number of vertical mirrors are generated by the 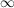 -fold axis. One twofold rotation axis is depicted and again there are infinitely many twofolds generated by the 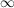 -fold axis. A series of events in the form of a blue ring (a flock of birds forming a ring?) in the upper and lower hemispheres is shown in panel Fig. 13 ▸(*c*) reflecting the 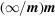 symmetry.

**Figure 14:**
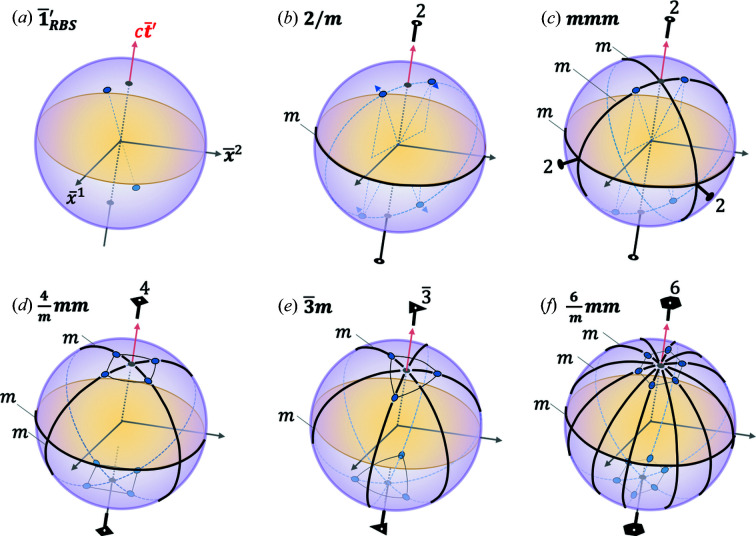
Six 3D holohedral RBS point groups for periodic RBS crystals. The sphere from Fig. 13 ▸(*c*) for the case of 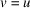 is shown in each panel, with appropriately placed events (blue ovals) on each hemisphere to break specific symmetries and retain others. The blue arrows associated with the events in panel (*b*) suggest a series of additional events stretching in the direction of the arrows. The generating symmetry elements for each group are indicated.

Existing symmetries of the isotropic 3D RBS can be broken by arranging various events in the 3D RBS so as to break certain symmetries and create RBS crystals with lower symmetry. The following Curie subgroups of 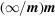 are also valid groups describing 3D RBS if some symmetries are broken: 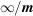 , 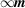 , 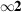 and 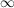 (Newnham, 2005 ▸). For example, by placing a single event in the upper hemisphere in Fig. 13 ▸(*a*) or 13 ▸(*b*) and nowhere else would break all the symmetries depicted in Fig. 13 ▸(*c*); it would correspond to the 3D point group labeled **1** whose only element is identity, 1. By placing two events, one related to the other by 3D RBS inversion, 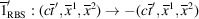 , one obtains the 3D RBS group 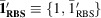 as shown in Fig. 14 ▸(*a*) for the 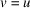 case from Fig. 13 ▸(*b*).

The 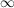 -fold axis can be replaced by a *p*-fold rotation (*p* is a natural number) using appropriately placed events. If one restricts themselves to periodic 3D space crystals, only one-, two-, three-, four- or sixfold rotation axes are allowed (Newnham, 2005 ▸). Fig. 14 ▸ shows events placed as blue ovals on the surface of an RBS surface for the 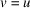 case [shown in Fig. 13 ▸(*b*)] in order to create six of the seven holohedral point groups in periodic 3D space crystals now applied to 3D RBS: 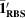 , **2/*m***, ***mmm***, **4/*mmm***, 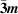 and **6/*mmm***. [The only missing holohedral group in Fig. 14 ▸ is the cubic group ***m*3*m*** which is not consistent with the 3D RBS. This is because in breaking symmetry through the placement of events, some aspects of the RBS are ‘baked in’ and cannot be changed, such as the RBS light lines, planes and cones, and the resulting ‘crease’ between the time-like and space-like events as seen in Fig. 13 ▸(*a*)]. All other RBS point groups are subgroups of these six RBS holohedral groups (Newnham, 2005 ▸). None of the 14 conventional colored 3D Curie groups listed by Newnham (2001 ▸) can be associated with the 3D RBS structures in Fig. 14 ▸ by the inclusion of 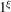 . Since 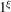 results in ‘dissolving’ and ‘reforming’ the light cones, and the crease between time-like and space-like event surfaces in Fig. 13 ▸(*a*), it does not conform to the definition of a typical symmetry element where no cuts or stitches to the object in question are allowed; that is the domain of topological distortions, and hence not discussed further here.

One can construct similar 4D RBS structures and the corresponding Curie groups. All the point groups and space groups for space crystals in 4D are listed in the literature (Brown *et al.*, 1978 ▸). The group 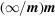 in 4D would be valid, except *m* would represent a hyperplane (of dimension 3) in 4D. For the case of 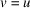 for the 4D 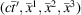 coordinates, the horizontal 4D hyperplane mirror perpendicular to the 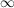 -fold rotation axis will be given by the diagonal tensor [−1, 1, 1, 1] (which is equivalent to the RBS time reversal in 4D). One of the vertical 4D hyperplane mirrors parallel to the 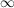 -fold axis would be, for example, the diagonal matrix given by [1, −1, 1, 1] perpendicular to the 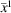 axis. The 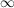 -fold axis parallel to the 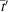 axis would rotate the stated vertical hyperplane mirror to generate infinitely many of them. The subgroups of this group would again be valid descriptions of the RBS. Crystallographic 4D RBS groups can also be deduced from the well enumerated 4D space crystallographic groups listed in the literature (Brown *et al.*, 1978 ▸).

## 10. Periodic RBS crystals   

The defining feature of periodic spatial crystals is their translational symmetry, namely, that they are periodic in various spatial dimensions. In describing their symmetry, one moves beyond point groups to add translations to create space groups (Glazer & Burns, 2013 ▸; Hahn, 2016 ▸). In the context of conventional MS, one moves from Lorentz groups to Poincaré groups. The group theoretical procedure to move from point groups to space groups is well established (Glazer & Burns, 2013 ▸). Here, given the equivalence established between space crystals and RBS crystals, one could similarly move from the RBS point groups to RBS Poincaré groups in analogy with space groups. Below, we limit our discussion to 2D, but similar extensions will be possible in higher dimensions.

**Figure 15:**
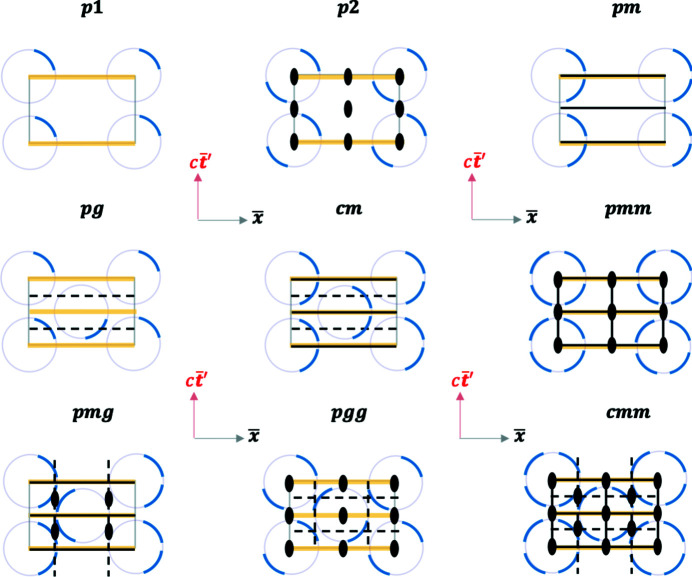
Examples of 2D RBS space groups adopting the same labels as the corresponding 2D space groups for space crystals. A unit cell is shown in each case by a gray rectangle. The faded purple circle at each lattice site is the same as the RBS circle in Fig. 9 ▸(*b*) for the case of 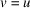 . The blue arcs represent a series of events (an RBS spacetime flock of birds?) being observed in RBS coordinates as indicated by the axes 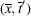 . Dashed black lines are glide planes, solid black lines are mirrors, yellow lines are light lines, and the black ovals represent twofold rotation axes.

There are 17 2D space-group types describing spatial crystals (Cotton, 1990 ▸). In order to keep the discussion simple, let us focus on the simplest case of 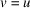 depicted by the 2D RBS group depicted in Fig. 9 ▸(*b*), where the blended coordinates are between the GF and the event BF. Since the light line is parallel to the space axis, 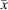 in this case, and the resulting symmetry as seen before is ***mm*2**, let us restrict our discussion to space groups whose site symmetries (point-group symmetries at individual locations within the crystal) are restricted to point-group symmetries of ***mm*2** or its subgroups. Fig. 15 ▸ shows such 2D RBS space groups, where the group labels are picked to be synonymous with the corresponding 2D space-group labels for space crystals.

The RBS crystals can be imagined as a series of events periodically arranged in the RBS being observed by an RBS observer at the origin. In the 2D case, the periodicity arises from translations along the 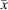 and the 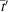 axes. Naturally, the event periodicity will result in the RBS observer herself being replicated periodically in the RBS as depicted. Glide planes (dashed lines in Fig. 15 ▸) can be observed now where one mirrors across the glide plane, and then translates by half a unit cell along the glide plane. These types of symmetries are not obvious in the conventional MS constructions of spacetime depicted in Fig. 2 ▸.

How about 2D space groups with say three-, four- and sixfold rotations? These are excluded in the case of a fixed relative orientation of the light lines in the RBS; higher-fold rotations than twofold will rotate the RBS light lines as well, and hence these RBS space groups will have to be composed with varying *v* in the RBS. Similar constructions can be made in 3D and 4D RBS. These are interesting topics left to be explored in future works.

## 11. Conclusion   

In conclusion, while time crystals are of great current interest (Shapere & Wilczek, 2012 ▸; Wilczek, 2012 ▸), this work extends the concept to relativistic spacetime crystals. By considering blended inertial frames between two inertial observers and then renormalizing the coordinates of an event observed by them by 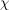 [which is a function given in equation (8) of the relative velocity between the ground and the train frames, *v*, and between the ground and the event frames, *u*], one can generate the RBS coordinates ( 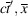 ) and ( 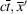 ). These coordinates transform the hyperbolic geometry of the Minkowski spacetime (MS) into a renormalized blended spacetime (RBS) that exhibits a Euclidean construction. The Lorentz boosts become continuous Euclidean rotations, and the RBS geometry also exhibits a new set of light lines. Mapping between the MS and the RBS frames shows that they have equivalent relativistic physics content: every point not on the light lines in MS maps to a unique point in RBS. Every point on the light lines in MS maps to the origin in RBS. Conversely, the light lines in the RBS map to the 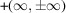 or 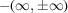 limits of the light lines in the MS. Points not on the light lines in the RBS uniquely map to points in the MS.

These mappings between MS and RBS give rise to equivalent representations of the relativistic physics in both descriptions. This is based on three considerations: (i) the equivalence mapping in Fig. 10 ▸ between the MS and RBS coordinates. (ii) Einstein’s first and second postulates hold still. Blending of the frames does not modify them, since one can always revert back from the RBS to the MS coordinates and recover these postulates. (iii) Lorentz transformation [equation (3)] and the invariance of the spacetime interval 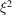 [equation (2)] are still valid, since the equivalent RBS statements in equations (11*c*)[Disp-formula fd11c] and (12), respectively, were derived from them.

However, mathematically speaking, the Euclidean geometry in RBS allows one to smoothly mathematically ‘cross’ the RBS light lines, which is not possible in the hyperbolic geometry in the MS. This feature allows us to write Lorentz boosts as Euclidean rotations, which in turn helps map the Lorentz group of the RBS to equivalent crystallographic symmetry groups already well known in space crystals. The RBS point groups in 2D, 3D and 4D are identified to be those associated with cylinders in various dimensions: rectangle in 2D, cylinder in 3D and hypercylinder in 4D. With the addition of translations, examples are given for 2D RBS space groups that describe RBS crystals; RBS space groups of higher dimensions should be straightforward in a similar manner. A *Mathematica* file is provided in the supporting information for a reader to plot the MS and RBS constructions for themselves.

On a more general mathematical note, this approach could allow one to straddle between Euclidean and hyperbolic coordinate systems in flat space or spacetime. For a set of *n* linearly independent coordinates 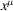 , 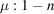 , if the eigenvalue of the metric tensor for the first *k* coordinates is −1, and that for the remaining ( 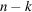 ) coordinates is +1, and if a linear transformation between 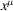 and 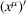 coordinates exists that leaves the interval 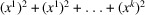 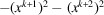 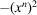 invariant before and after the transformation, then one can define a blended coordinate system between primed and unprimed coordinates with a Euclidean interval 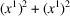 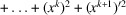 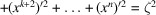 . If 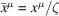 is defined, then 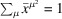 is a unit circle in a Euclidean frame. Going forward, it will be interesting to explore quasi-1D RBS magnetic groups, periodic and aperiodic RBS crystallographic groups in various dimensions, RBS quasicrystals, and the full scope of the renormalized blended frames in covariant electrodynamics, relativistic physics and quantum gravity. Appendix *A* [App appa] provides a preliminary sketch for how one might consider extensions of this work to general relativity.

## Supplementary Material

Mathematica notebook to generate the spacetime plots in figures 2-9 given in the article. DOI: 10.1107/S2053273321003259/ib5098sup1.nb

A PDF of the output generated by the Mathematica notebook file. DOI: 10.1107/S2053273321003259/ib5098sup2.pdf

## Appendix A Sketch of blended coordinates in the Rindler and Schwarschild geometries

The line element in Rindler geometry in a flat 2D spacetime is given by the differential line element  =  =  which captures many of the same properties as the Schwarzschild geometry in general relativity (Dray, 2015 ▸). The second equality uses the hyperbolic polar coordinates (  ) shown in Fig. 2 ▸, which are also called the Rindler coordinates. Upon computing  in the train inertial frame (TF) moving at a constant relative speed of  with respect to the ground frame (GF), one can show that  , since  is assumed constant; thus,  is an invariant.

If we now define a Rindler differential line element in the blended frame as  , then one can show that  , where  . Both the factors  and  are functions of  which determines the relative speeds of the two inertial frames (GF and TF).

Consider two special cases in the Rindler geometry above: a constant acceleration (  ), and (trivially) a constant velocity (  ) of the bird. In the former case (  ),  , and thus one could define renormalized blended coordinates  =  and  , such that  , a unit circle for any worldline in the Rindler geometry with a constant acceleration. In the latter case (  ,  , and one could define renormalized blended coordinates  and  , to again recover a unit circle. More generally, one could define  and  , such that  for any worldline in the Rindler geometry.

Now let us consider the curved spacetime. The Schwarzchild metric describes the gravitational field of a point mass, *m*, at the origin; it is a spherically symmetric solution of Einstein’s equation in vacuum (Dray, 2015 ▸). The line element is given in polar coordinates,  , with the origin centered at the mass by

where  and  , where the abbreviation  and  has been used. As a specific example, consider a shell observer sitting on an imaginary shell at a radius *r* from the mass, on the equator at a fixed  (  and a fixed azimuth (  . Note that as  , this metric reduces to that of the Minkowski metric of flat spacetime. Consider the rain coordinates of a radial geodesic (for example, a radial worldline from  towards  ). The relative speed of the radial observer as she crosses the shell observer can be shown to be  , where the minus sign indicates motion in the  , or the inward radial direction. A Lorentz transformation between the shell coordinates (  ) and the rain coordinates (  ) is given by  , where

Further, the metric is invariant, namely,  =  . If we now consider a blended reference frame between the shell and the rain coordinates, then  =  . Rearranging, we can rewrite this as  +  , where  and  . Thus, in principle, blended renormalized Euclidean coordinates are possible locally on a manifold in general relativity.
