# Supplementary material for: Relativistic spacetime crystals
Source: Acta Crystallogr A Found Adv. 2021 May 27;77(Pt 4):242–56. doi: 10.1107/S2053273321003259 (PMC8248891; doi:10.1107/S2053273321003259)
Supplement: Supplementary file 2 [file a-77-00242-sup2.pdf]

FOUNDATIONS
ADVANCES

Volume 77 (2021)

Supporting information for article:

Relativistic Spacetime Crystals

Venkatraman Gopalan

Relativistic Blended Spacetime Crystals, V. Gopalan

Comments on Figure 1

```
(* Figure 1:
 Definitions of different inertial frames and observers.
    Defines a ground frame (GF) observer
    A train frame (TF) observer moving
   at speed v relative to the GF in the +x direction.
    A bird (an event) being observed by the GF and TF observers,
moving at a speed u with respect to the GF in the +x direction.
    A frame moving with the bird
   is called the bird frame (BF) or the proper frame.
    The hyperbolic angles are defined as:
    Tanh(α)=v/c
    Tanh(β)=u/c  *)
```

Comments on Figure 2

```
(*Figure 2:
     2D Minkowski spacetime (MS) coordinates
        are plotted in hyperbolic geometry. The code below plots..
        the hyperbolic branch F as ξ{Sinh[β],Cosh[β]}, ξ=1
        the hyperbolic branch P as ξ{-Sinh[β],-Cosh[β]}, ξ=1
        the hyperbolic branch U as ξ{Cosh[β],Sinh[β]}, ξ=1
        the hyperbolic branch T as ξ{-Cosh[β],-Sinh[β]}, ξ=1
        the light line x=ct as {ξ,ξ}
        the light line x=-ct as {ξ,-ξ}
        the coordinates of the ct'
        axis is {ξ*Sinh[ArcTanh[0.9]],ξ*Cosh[ArcTanh[0.9]]}
        the coordinates of the x' axis is {ξ*Cosh[ArcTanh[0.9]],
         ξ*Sinh[ArcTanh[0.9]]}}
       the plotting range for the hyperbolic angle β is -ArcTanh[0.999]<
 β<ArcTanh[0.999], which corresponds to -0.999c<v<0.999c
       the plotting range for the spacetime interval ξ is -5 <ξ<5 *)
```

```
Clear[β, ξ]
ParametricPlot[{{Sinh[β], Cosh[β]},
  {-Sinh[β], -Cosh[β]}, {Cosh[β], Sinh[β]}, {-Cosh[β], -Sinh[β]},
  {ξ, ξ}, {ξ, -ξ}, {ξ * Sinh[ArcTanh[0.9]], ξ * Cosh[ArcTanh[0.9]]},
  {ξ * Cosh[ArcTanh[0.9]], ξ * Sinh[ArcTanh[0.9]]}},
 {β, -ArcTanh[0.999], ArcTanh[0.999]}, {ξ, -3, 3}, PlotRange → {{-3, 3}, {-3, 3}}]
```

Code for Figure 2

Out[46]=

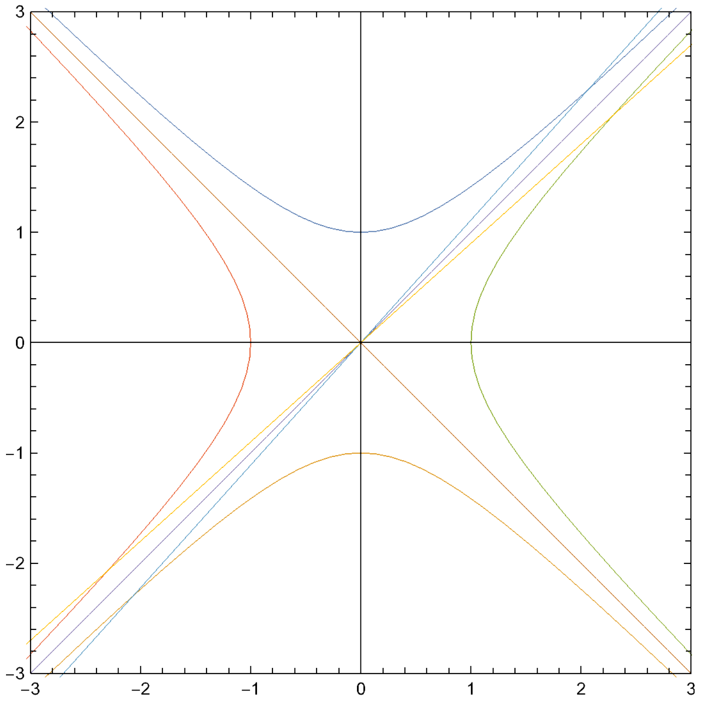

Figure 2

Comments on Figure 3

```
(*Figure 3:
    A plot of the renormalization factor,
χ=√(Abs[Sec[θ]*Sec[2*ϕ-θ]]) from Eq. (8) as a function of u/c = sinϕ/cos(θ-ϕ).
    By construction, v/c = sinθ.  Three different cases for v are explored.:
    (1) θ=ArcSin[0], corresponding to v=0
    (2) θ=ArcSin[0.9], corresponding to v=0.9c
    (3) θ=ϕ, corresponding to v=u
   The range over which ϕ is plotted is ArcSin[-0.99999]<ϕ<ArcSin[0.99999],
which corresponds to -0.99999c<u<0.99999c *)
```

```
Clear[θ, ϕ, ξ]
θ = ArcSin[0]
p1 = ParametricPlot[{{Sin[ϕ] / Cos[ϕ - θ], √(Abs[Sec[θ] * Sec[2 * ϕ - θ]])}},
  {ϕ, ArcSin[-0.99999], ArcSin[0.99999]}, PlotRange → {{-1, 1}, {0, 10}}]
θ = ArcSin[0.9]
p2 = ParametricPlot[{{Sin[ϕ] / Cos[ϕ - θ], √(Abs[Sec[θ] * Sec[2 * ϕ - θ]])}},
  {ϕ, ArcSin[-0.99999], ArcSin[0.99999]}, PlotRange → {{-1, 1}, {0, 10}}]
θ = ϕ
p3 = ParametricPlot[{{Sin[ϕ] / Cos[ϕ - θ], √(Abs[Sec[θ] * Sec[2 * ϕ - θ]])}},
  {ϕ, ArcSin[-0.99999], ArcSin[0.99999]}, PlotRange → {{-1, 1}, {0, 10}}]
Show[p1, p2, p3, PlotTheme → "Minimal"]
```

Out[ ]= 0

Code for Figure 3

Out[∘]=

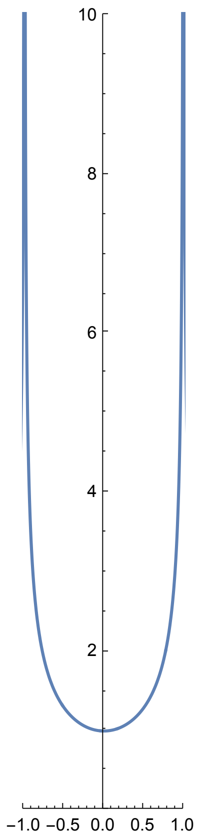

Figure 3b

Out[∘]= 1.11977

Out[∘]=

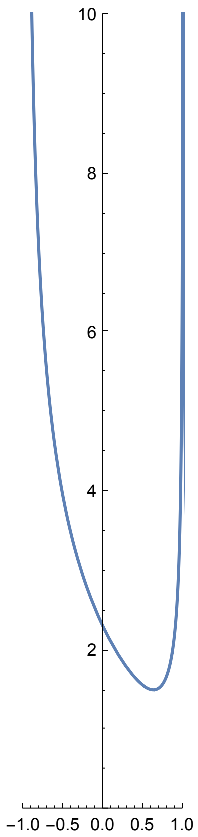

Figure 3c

Out[∘]= ϕ

Out[ ]=

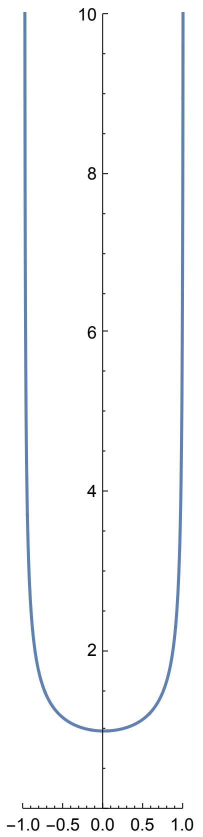

Figure 3a

Out[ ]=

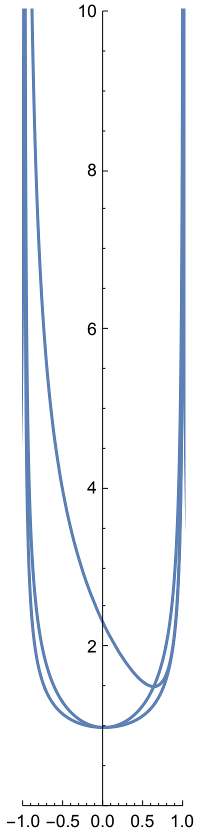

Figure 3a, b, c

Comments on Figure 4

```
(*Figure 4
      2D Blended Spacetime coordinates
  (x, ct') are plotted from Eq. (7) for ξ=1 :
      x=ξ*χ*Sin[ϕ]
      ct'=ξ*χ*Cos[ϕ]

     The factor χ= √(Abs[Sec[θ]*Sec[2*ϕ-θ]]) is substituted from Eq (8)
     The specific case of θ=0 is assumed, which corresponds to v=0.  *)
```

```
Clear[θ, ϕ, ξ]
θ = ArcSin[0]
ξ = 1
χ = √(Abs[Sec[θ] * Sec[2 * ϕ - θ]])
x = ξ * χ * Sin[ϕ]
tp = ξ * χ * Cos[ϕ]
ParametricPlot[{{x, tp}, {ξ, ξ}, {ξ, -ξ}}, {ϕ, -Pi, Pi},
 {ξ, -5, 5}, PlotPoints → 50, PlotRange → {{-3, 3}, {-3, 3}}]
```

Code for Figure 4

Out[55]= 0

Out[56]= 1

Out[57]= $\sqrt{\text{Abs}[\text{Sec}[2\,\phi]]}$

Out[58]= $\sqrt{\text{Abs}[\text{Sec}[2\,\phi]]}\ \text{Sin}[\phi]$

Out[59]= $\sqrt{\text{Abs}[\text{Sec}[2\,\phi]]}\ \text{Cos}[\phi]$

Out[60]=

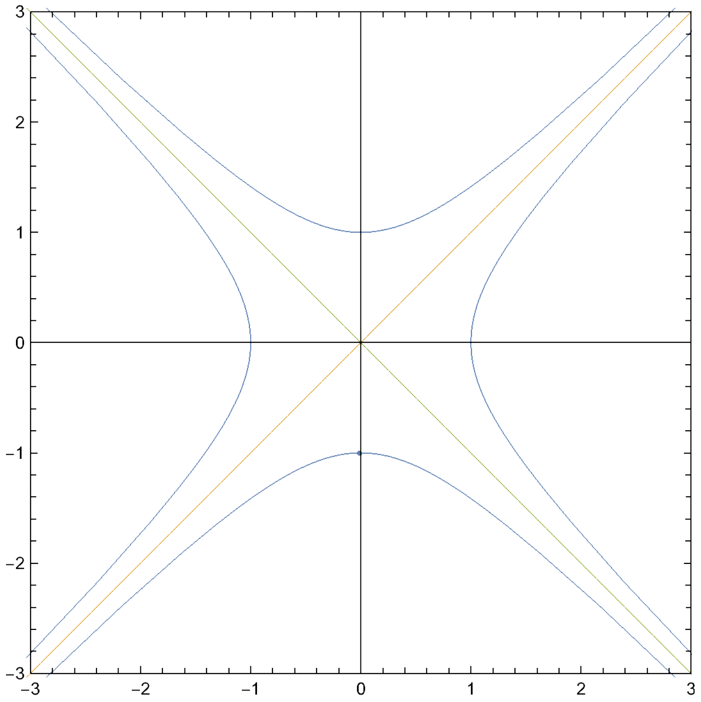

Figure 4

Comments on Figure 5

```
(*Figure 5
      2D Blended Spacetime
  coordinates (x, ct') is plotted from Eq. (7) for ξ=1 :
      x=ξ*χ*Sin[ϕ]
      ct'=ξ*χ*Cos[ϕ]

      The factor χ= √(Abs[Sec[θ]*Sec[2*ϕ-θ]]) is substituted from Eq (8)
      The specific case of θ=ArcSin[0.9] is assumed,
which corresponds to v=0.9c  *)
```

```
Clear[θ, ϕ, ξ]
θ = ArcSin[0.9]
ξ = 1
χ = √(Abs[Sec[θ] * Sec[2 * ϕ - θ]])
x = ξ * χ * Sin[ϕ]
tp = ξ * χ * Cos[ϕ]
ParametricPlot[{{x, tp}, {ξ, ξ * 0.229416}, {ξ, -ξ * 4.358898943540674`}},
 {ϕ, -Pi, Pi}, {ξ, -5, 5}, PlotRange → {{-5, 5}, {-5, 5}}]
```

```
Out[90]= 1.11977
Out[91]= 1
Out[92]= 1.51465 √(Abs[Sec[1.11977 - 2 ϕ]])
Out[93]= 1.51465 √(Abs[Sec[1.11977 - 2 ϕ]]) Sin[ϕ]
Out[94]= 1.51465 √(Abs[Sec[1.11977 - 2 ϕ]]) Cos[ϕ]
```

Code for Figure 5

Out[95]=

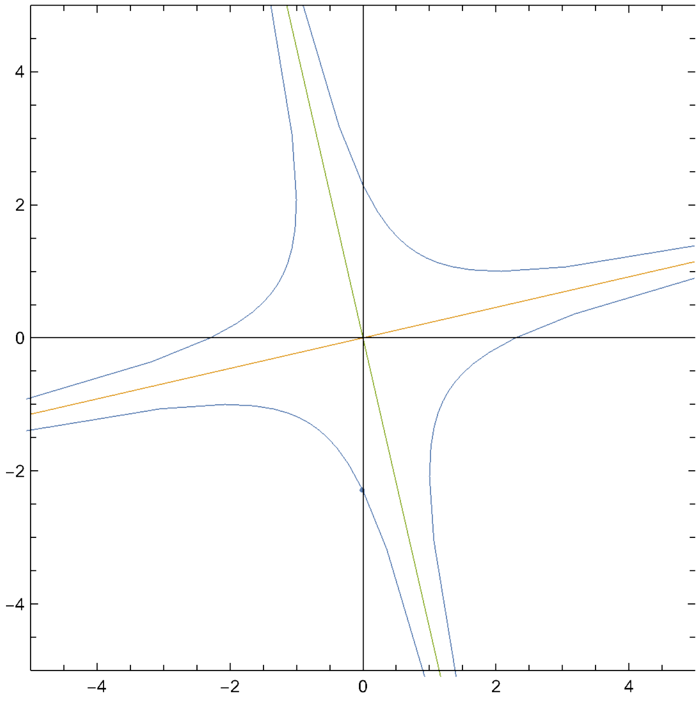

Figure 5

Comment on Figure 6

```
(*Figure 6
  2D RBS coordinates (xn, tpn) from Eq. (11) are plotted for ξ=1 :
   xn=χ*Sin[ϕ]/χ
    tpn=χ*Cos[ϕ]/χ
     The factor χ= √(Abs[Sec[θ]*Sec[2*ϕ-θ]]) is substituted from Eq (8)
      The specific case of θ=ArcSin[0] is assumed, which corresponds to v=0
   Light line x=ct is plotted as {ξ,ξ}
    Light line x=-ct is plotted as {ξ,-ξ}  *)
```

```
Clear[θ, ϕ, ξ, χ, x, tp]
θ = ArcSin[0]
ξ = 1
χ = √(Abs[Sec[θ] * Sec[2 * ϕ - θ]])
xn = χ * Sin[ϕ] / χ
tpn = χ * Cos[ϕ] / χ
ParametricPlot[{{xn, tpn}, {ξ, ξ}, {ξ, -ξ}},
 {ϕ, -Pi, Pi}, {ξ, -2, 2}, PlotRange → {{-2, 2}, {-2, 2}}]
```

Code for Figure 6

Out[83]= 0

Out[84]= 1

Out[85]= $\sqrt{\text{Abs}[\text{Sec}[2\,\phi]]}$

Out[86]= Sin[ϕ]

Out[87]= Cos[ϕ]

Out[88]=
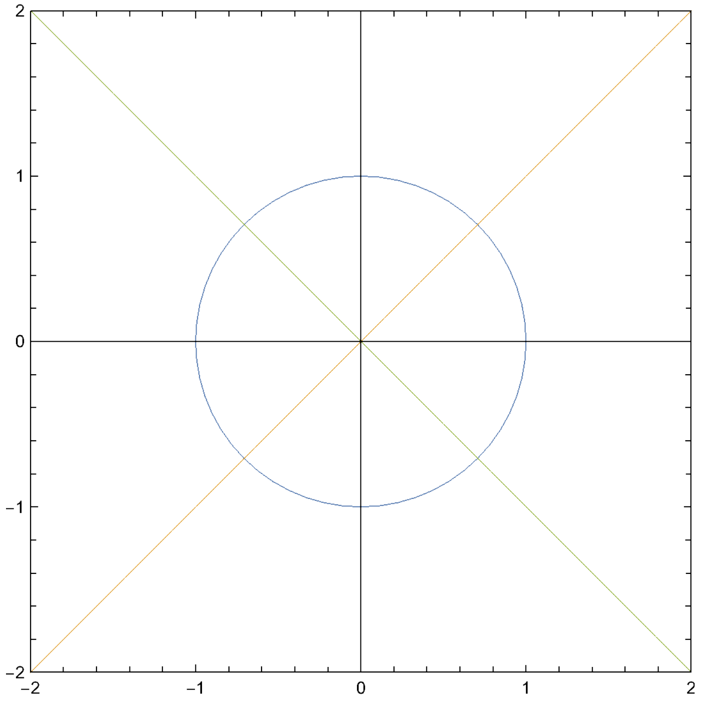

Figure 6

Comments for Figure 7

```
(*Figure 7
  2D RBS coordinates (xn, tpn) from Eq. (11) are plotted for ξ=1 :
   xn=χ*Sin[ϕ]/χ
    tpn=χ*Cos[ϕ]/χ
     The factor χ= √(Abs[Sec[θ]*Sec[2*ϕ-θ]]) is substituted from Eq (8)
      The specific case of θ=ArcSin[0.9] is assumed,
which corresponds to v=0.9c
   Light line x=ct is plotted as ~{ξ,0.229ξ}
    Light line x=-ct is plotted as ~{ξ,-4.3589ξ}
     The slopes of the light lines were found by finding the solutions to the
     equations x=ct and x=-ct using the coordinates in Eqs. (7) and (11)
      In particular, sinϕ=cos(ϕ-θ) gives one of the light lines,
and cosϕ=sin(ϕ-θ) gives the other light line. *)
```

```
Clear[θ, ϕ, ξ, χ, x, tp]
θ = ArcSin[0.9]
ξ = 1
χ = √(Abs[Sec[θ] * Sec[2 * ϕ - θ]])
xn = χ * Sin[ϕ] / χ
tpn = χ * Cos[ϕ] / χ
ParametricPlot[{{xn, tpn}, {ξ, ξ * 0.229416}, {ξ, -ξ * 4.358898943540674`}},
 {ϕ, -Pi, Pi}, {ξ, -2, 2}, PlotRange → {{-2, 2}, {-2, 2}}]
```

Out[97]= 1.11977

Code for Figure 7

Out[98]= 1

Out[99]= 1.51465 $\sqrt{\text{Abs}[\text{Sec}[1.11977 - 2\,\phi]]}$

Out[100]= 1. Sin[ϕ]

Out[101]= 1. Cos[ϕ]

Out[102]=

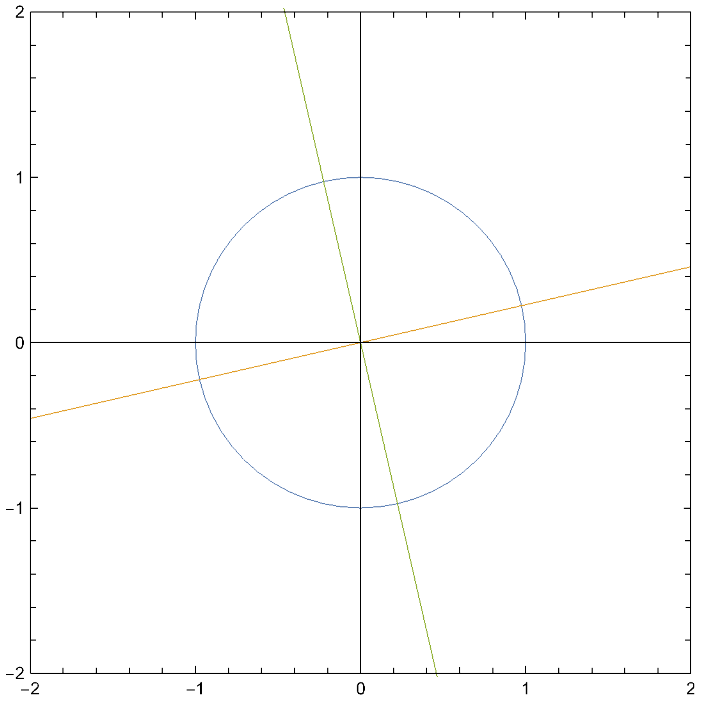

Figure 7

In[166]:=

Comments for Figure 8a

```
(*Figure 8a
  2D Blended Spacetime coordinates (x, ct') is plotted from Eq. (7) for ξ=1 :
      x=ξ*χ*Sin[ϕ]
      ct'=ξ*χ*Cos[ϕ]

    The factor χ=√(Abs[Sec[θ]*Sec[2*ϕ-θ]]) is substituted from Eq (8)
    The specific case of θ=ArcSin[0.99999] is assumed,
which corresponds to v=0.99999c, that is v → c infinitesimally closely. *)
```

```
Clear[θ, ϕ, ξ]
θ = ArcSin[0.99999]
ξ = 1
χ = √(Abs[Sec[θ] * Sec[2 * ϕ - θ]])
x = ξ * χ * Sin[ϕ]
tp = ξ * χ * Cos[ϕ]
ParametricPlot[{{x, tp}}, {ϕ, -Pi, Pi}, PlotRange → {{-40, 40}, {-40, 40}}]
```

Out[167]= 1.56632

Code for Figure 8a

Out[168]= 1

Out[169]= $14.9535 \sqrt{\text{Abs}[\text{Sec}[1.56632 - 2\,\phi]]}$

Out[170]= $14.9535 \sqrt{\text{Abs}[\text{Sec}[1.56632 - 2\,\phi]]}\ \text{Sin}[\phi]$

Out[171]= $14.9535 \sqrt{\text{Abs}[\text{Sec}[1.56632 - 2\,\phi]]}\ \text{Cos}[\phi]$

Out[172]=

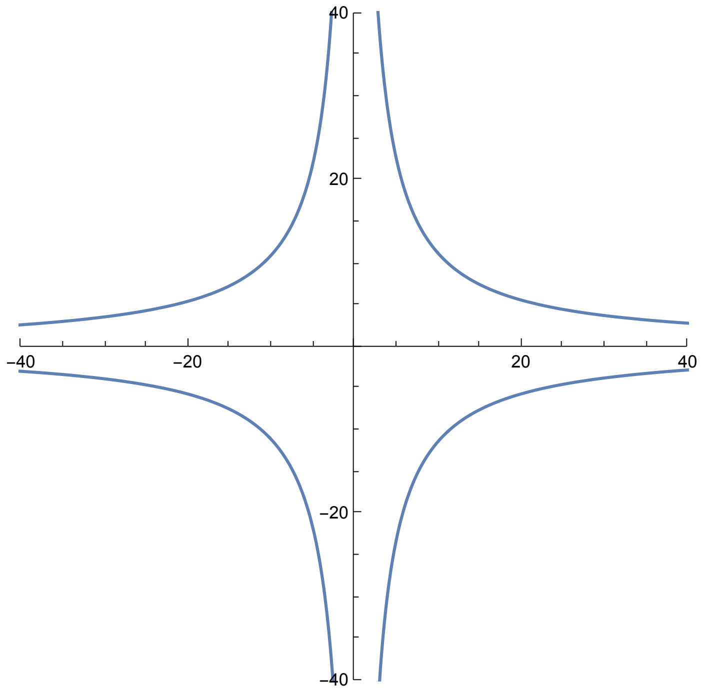

Figure 8a

Comments for Figure 8b

```
(*Figure 8b
  2D RBS coordinates (xn, tpn) from Eq. (11) are plotted for ξ=1 :
   xn=χ*Sin[ϕ]/χ
    tpn=χ*Cos[ϕ]/χ
     The factor χ=√(Abs[Sec[θ]*Sec[2*ϕ-θ]]) is substituted from Eq (8)
      The specific case of θ=ArcSin[0.99999] is assumed,
which corresponds to v=0.99999c,  that is v → c infinitesimally closely.
   Light line x=ct approaches infinitesimally close to the x-axis
    Light line x'=-ct' approaches infinitesimally close to the ct'-axis *)
```

```
Clear[θ, ϕ, ξ, χ, x, tp]
θ = ArcSin[0.99999]
ξ = 1
χ = √(Abs[Sec[θ] * Sec[2 * ϕ - θ]])
xn = χ * Sin[ϕ] / χ
tpn = χ * Cos[ϕ] / χ
ParametricPlot[{{xn, tpn}}, {ϕ, -Pi, Pi}, PlotRange → {{-2, 2}, {-2, 2}}]
```

Out[132]= 1.56632

Code for Figure 8b

Out[133]= 1

Out[134]= $14.9535 \sqrt{\text{Abs}[\text{Sec}[1.56632 - 2\,\phi]]}$

Out[135]= 1. Sin[ϕ]

Out[136]= 1. Cos[ϕ]

Out[137]=

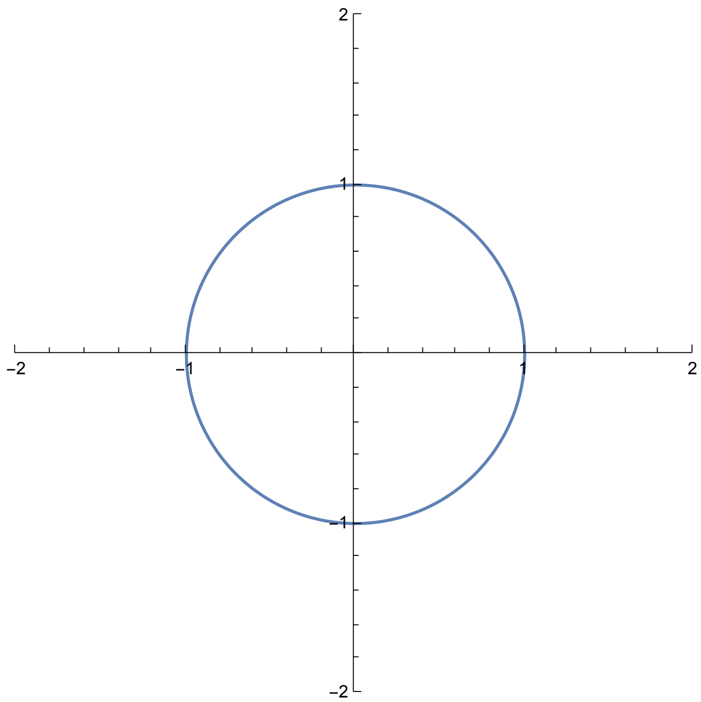

Figure 8b

Comments for Figure 9a

```
(*Figure 9a
  2D Blended Spacetime coordinates (x, ct') is plotted from Eq. (7) for ξ=1 :
       x=ξ*χ*Sin[ϕ]
       ct'=ξ*χ*Cos[ϕ]

     The factor χ=√(Abs[Sec[θ]*Sec[2*ϕ-θ]]) is substituted from Eq (8)
     The specific case of θ=ϕ is assumed, which corresponds to v=u *)
```

```
Clear[θ, ϕ, ξ]
θ = ϕ
ξ = 1
χ = √(Abs[Sec[θ] * Sec[2 * ϕ - θ]])
x = ξ * χ * Sin[ϕ]
tp = ξ * χ * Cos[ϕ]
ParametricPlot[{{x, tp}}, {ϕ, -Pi, Pi}, PlotRange → {{-5, 5}, {-1.5, 1.5}}]
```

Out[188]= ϕ

Code for Figure 9a

Out[189]= 1

Out[190]= Abs[Sec[ϕ]]

Out[191]= Abs[Sec[ϕ]] Sin[ϕ]

Out[192]= Abs[Sec[ϕ]] Cos[ϕ]

Figure 9a

Out[193]=

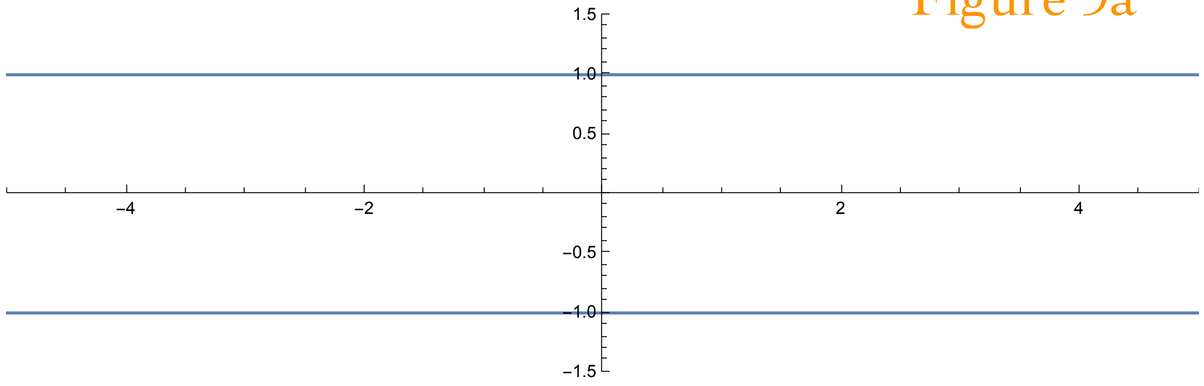

Comments for Figure 9b

```
(*Figure 9b
  2D RBS coordinates (xn, tpn) from Eq. (11) are plotted for ξ=1 :
   xn=χ*Sin[ϕ]/χ
    tpn=χ*Cos[ϕ]/χ
     The factor χ=√(Abs[Sec[θ]*Sec[2*ϕ-θ]]) is substituted from Eq (8)
      The specific case of θ=ϕ is assumed,
which corresponds to v=u,  that is, the TF and the BF are coincident.
   Light lines x=+/-ct  are parallel to the x-axis. *)
```

```
Clear[θ, ϕ, ξ, χ, x, tp]
θ = ϕ
ξ = 1
χ = √(Abs[Sec[θ] * Sec[2 * ϕ - θ]])
xn = χ * Sin[ϕ] / χ
tpn = χ * Cos[ϕ] / χ
ParametricPlot[{{xn, tpn}}, {ϕ, -Pi, Pi}, PlotRange → {{-2, 2}, {-2, 2}}]
```

Code for Figure 9b

Out[195]= ϕ

Out[196]= 1

Out[197]= Abs[Sec[ϕ]]

Out[198]= Sin[ϕ]

Out[199]= Cos[ϕ]

Out[200]=

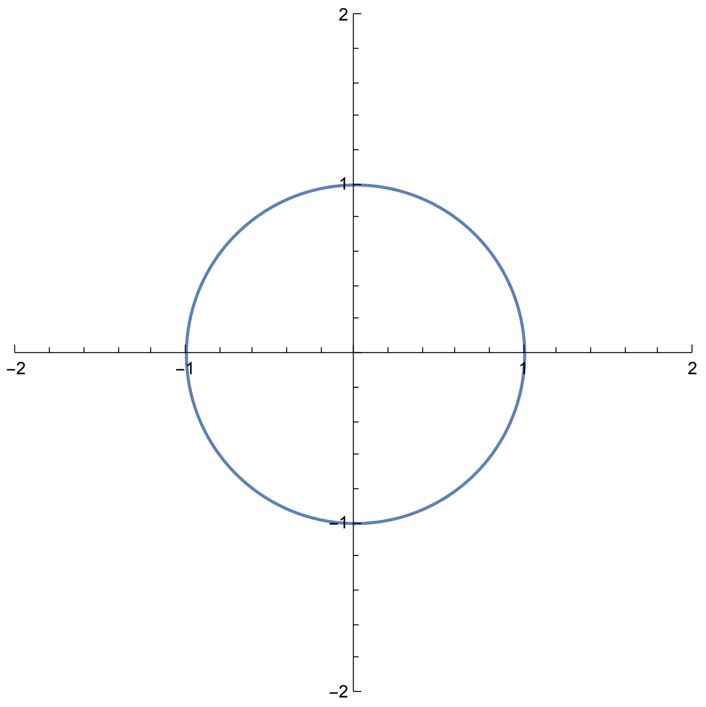

Figure 9b
